# Supplementary material for: Immune and metabolic markers for identifying and investigating severe Coronavirus disease and Sepsis in children and young people (pSeP/COVID ChYP study): protocol for a prospective cohort study
Source: BMJ Open. 2023 Mar 27;13(3):e067002. doi: 10.1136/bmjopen-2022-067002 (PMC10069273; doi:10.1136/bmjopen-2022-067002)
Supplement: Supplementary data [file bmjopen-2022-067002supp001.pdf]

For each patient, free text field 300-400 words.

Once data are input, can be reedited in line with new diagnosis.

Research ID number					
Age (in years and months)	*To encode formula to calculate age upon input of date of birth (for confidentiality)*				
Age	<1	2-5	6-12	13-15	16-17
Sex	M	F	Indeterminate		
Ethnicity	White	Black	West Asian		
	East Asian	South Asian			
	Other – please specify				
Weight (kgs)					
Weight centile	< 0.4<br>0.4 to 2<br>2 to 9<br>9 to 75<br>75 to 91<br>91 to 99.6<br>>99.6				

Inclusion Criteria

Admitted to PCCU<br>(Or)<br>Admitted to ward with confirmed COVID positive and/or PIMS-Ts	Yes/No
Acute illness including trauma	Yes/No
Routine blood tests	Yes/No
Age <18 years	Yes/No

Exclusion Criteria

Planned admission to PCCU with no acute illness	Yes/No
Declined Consent for research<br>(Or)	Yes/No

Did not respond to follow up letter	
Language barrier cannot understand English/Welsh	Yes/No

Consent: Yes/No

(Please obtain consent before recording further details)

Co-morbidities: *If yes, please specify or give details regarding co-morbidity (prompt in eCRF)*

Congenital Heart disease Yes / No	Chronic Lung disease Yes/No	Neurological Problem Yes/No
Chronic renal disease Yes/No	Gastrointestinal Yes/No	Asthma Yes/No
Diabetes Yes/No	Liver disease Yes/No	Malignancy Yes/No
Expremature Yes/No	Haematological problem Yes/No	Immune disorder (including auto-immune and immune deficiency) Yes/No
Other: please specify		

History available for any vaccination in previous 4 weeks: Yes/No

If 'Yes', what vaccination? :

Acute illness onset timeline

Date of onset of symptoms	DD:MM:YY
Date of admission to hospital	DD:MM:YY
Date of admission to ward	DD:MM:YY
Date of admission to PCCU	DD:MM:YY

Please derive relation to research sample and other info such as microbiological tests and outcome variables

Brief history of illness and background

(*please* ***do not*** *include any patient identifiable information such as name, date of birth, age or address***)**

Admission signs and symptoms

Cough Yes/No	Respiratory distress Yes/No	Wheeze Yes/No	Apnoea Yes/No

Runny nose /Rhinorrhoea Yes/No	Sore throat Yes/No	Recessions Yes/No	Lower chest in drawing Yes/No
Grunting Yes/No	Head Bobbing Yes/No	Colour change Yes/No<br>• Specify:	Cardio Respiratory arrest Yes/No
Palpitations Yes/No	Arrhythmia Yes/no	Cool peripheries Yes/No	Thread /weak peripheral pulses Yes/No
Fever Yes/No	Chills/Rigors Yes/No	Seizure Yes/No	Abnormal movement Yes/No
Lethargy/weakness Yes/No	Headache Yes/No	Photophobia Yes/No	Neck stiffness Yes/No
Focal Neurology Yes/No	Poor tone Yes/No	Pupillary abnormalities Yes/No	Altered Consciousness Yes/No<br>• Irritable<br>• Agitation<br>• Abnormal behaviour<br>• Drowsy Yes/No
Vomiting Yes/No<br>• Bilious Vomiting Yes/No<br>• Bloody Vomiting Yes/No	Diarrhoea Yes/No	Bloody stools Yes/No	Constipation Yes/No
Abdominal distension Yes/No	Abdominal pain Yes/No	Reduced feeds Yes/No	Loss of appetite Yes/No
Weight Loss Yes/No	Skin Rash Yes/No<br>• Maculo/popular Yes/No<br>• Petechial/Ecchymosis Yes/No<br>• Vesicular Yes/No<br>• Other - specify	Lymphadenopathy Yes/No	Bleeding Yes/No
Reduced Urine Output Yes/No	Excessive Urination Yes/no	Haematuria Yes/No	Other Please specify

Admitted following unplanned/emergency surgery: Yes/No

If yes: please specify

PCCU Admission vital observations and laboratory details

FiO2(0.21 -1)	**Number 0.XX**
SpO2 %	**Number XXX**
HR beats/min	**NUMBER XXX**
RR Breaths/min	**NUMBER XXX**
BP mm of Hg<br>• Systolic<br>• Diastolic<br>• Mean	**NUMBER XXX**
Temp Centigrade<br>• Core<br>• Peripheral	**NUMBER XX.X**
GCS	**NUMBER XX**
Blood gas<br>• pH<br>• PaO2 KPa<br>• PaCO2 KPa<br>• Base Excess<br>• Bicarbonate mmol/L	**pH NUMBER X.XX**<br>**PaO2 NUMBER XX.XX**<br>**PaCO2 NUMBER XX.XX**<br>**BASE EXCESS NUMBER XX.XX**<br>**BICARBONATE NUMBER XX.X**
Urine output MLS/KG/HR	**NUMBER XX.XX**
Lactate mmol/L	**NUMBER XX.XX**
CRP mg/L	**NUMBER XXX.X**
Beta D Glucan pg/ml	**XXX.X**
Hb g/l	**XX.X**
WCC number / cumm<br>• Lymphocytes<br>• Neutrophils	**NUMBER XXX.X**
Platelets number/cumm	**NUMBER XXXX**
PT seconds	**NUMBER XX**
APTT second	**NUMBER XXX**
INR ratio	**NUMBER X.X**
ALT U/L	**NUMBER XXXXXX**
Bilirubin µmol/L	**NUMBER XX.X**
Urea mmol/L	**NUMBER XX.X**
Creatinine µmol/L	**NUMBER XXX.X**
Na mmol/L	**NUMBER XXX**
K mmol/L	**NUMBER XX.X**
Cl mmol/L	**NUMBER XXX.X**
d-Dimers µg/L	
Ferritin µg/L	**NUMBER XXXX**
CXR Signs of Lower Resp Infection	**Yes/No**
Other<br>• specify	

First observations since onset of acute illness on Ward / presentation to ED (please circle first if Ward or ED) from local or referring hospital.

FiO2(0.21 - 1	**Number 0.XX**
SpO2 %	**Number XXX**
HR beats/min	**NUMBER XXX**
RR Breaths/min	**NUMBER XXX**
BP mm of Hg<br>• Systolic<br>• Diastolic<br>• Mean	**NUMBER XXX**
Temp Centigrade<br>• Core<br>• Peripheral	**NUMBER XX.X**
GCS	**NUMBER XX**
Blood gas<br>• pH<br>• PaO2 KPa<br>• PaCO2 KPa<br>• Base Excess<br>• Bicarbonate mmol/L	**pH NUMBER X.XX**<br>**PaO2 NUMBER XX.XX**<br>**PaCO2 NUMBER XX.XX**<br>**BASE EXCESS NUMBER XX.XX**<br>**BICARBONATE NUMBER XX.X**
Urine output MLS/KG/HR	**NUMBER XX.XX**
Lactate mmol/L	**NUMBER XX.XX**
CRP mg/L	**NUMBER XXX.X**
Beta D Glucanpg/ml	**XXX.X**
Hb g/l	**XX.X**
WCC number / cumm<br>• Lymphocytes<br>• Neutrophils	**NUMBER XXX.X**
Platelets number/cumm	**NUMBER XXXX**
PT seconds	**NUMBER XX**
APTT second	**NUMBER XXX**
INR ratio	**NUMBER X.X**
ALT U/L	**NUMBER XXXXXX**
Bilirubin µmol/L	**NUMBER XX.X**
Urea mmol/L	**NUMBER XX.X**
Creatinine µmol/L	**NUMBER XXX.X**
Na mmol/L	**NUMBER XXX**
K mmol/L	**NUMBER XX.X**
Cl mmol/L	**NUMBER XXX.X**
d-Dimers µg/L	
Ferritin µg/L	**NUMBER XXXX**
CXR Signs of Lower Resp Infection	**Yes / No**
Other<br>• specify	

Paediatric Index of Mortality (PIM-3) score: decimal. 0-1 (range)

Provisional Diagnosis

1. Provisional Diagnosis	**Comments multiple**
2. Provisional Diagnosis	

Organ Support in the first 24 hours

Respiratory Yes/No<br>If ‘Yes’ tick relevant box to select	• Non Invasive<br>○ High Flow<br>○ CPAP<br>○ BiPAP<br>• Invasive Mechanical ventilation<br>• High Frequency Oscillation	
Cardiovascular<br>Vaso active infusions Yes/No. If ‘Yes’ ...tick relevant box to select	• Dopamine<br>• Dobutamine<br>• Adrenaline<br>• Noradrenaline<br>• Vasopressin<br>• Milrinone<br>Other – please specify	
Haemofiltration/Haemodialysis Yes/No		
Extra corporeal life support Yes/No		
Worse PELOD score during illness		

1ST Research blood sample at admission

Date / Time to be entered on CRF: DD:MM:YYYY HH:MM

Suspected sepsis /suspected COVID/ non-septic illness

Daily vital observations and laboratory results (worst and best) - please can you make it easy for Awen and Iona to input data as this is data collected for every day of PICU admission of child?

Date of PCCU admission: _ _DD:MM:YY.

To derive relation to time of admission to PCCU, research sample, adverse events and microbiological sample results

FiO2(0.21 -1.0)<br>• highest<br>• lowest	**NUMBER .XX**
SpO2 %<br>• lowest<br>• highest	**NUMBER XXX**
HR beats/min<br>• highest<br>• lowest	**NUMBER XXX**
RR Breaths/min<br>• highest<br>• lowest	**NUMBER XX**
BP mm of Hg<br>• Systolic<br>Lowest in 24 hours<br>Highest in 24 hours<br>• Diastolic<br>Lowest in 24 hours<br>Highest in 24 hours<br>• Mean<br>Lowest in 24 hours<br>Highest in 24 hours	**NUMBER XXX**
Temp Centigrade<br>• Core<br>• Peripheral	**NUMBER XX.X**
GCS<br>lowest<br>highest	**NUMBER XX**

Blood gas (blood gas parameters in any sample for a 24 hours period)<br><br>• pH (lowest in 24 hrs)<br>• PaO2 KPa (lowest in 24 hours)<br>• PaCO2 KPa (highest in 24 hours)<br>• Base Excess (from the lowest pH gas result)<br>• Bicarbonate mmol/L (from the lowest pH gas result)	**NUMBERS**<br><br>**pH X.XX**<br>**PaO2 XX.X**<br>**PaCO2 XX.X**<br>**Base Excess XX.X**<br>**Bicarbonate XX.X**
Urine output (UOP)<br>Lowest UOP in any 6 hours over a 24 hours period<br>Diuretic medication Y/N	**NUMBER**<br>**XX.X**
Lactate mmol/L<br>Highest lactate	**NUMBER**<br>**XX.X**
CRP mg/L	**NUMBER XXX.X**
Beta D Glucan pg/ml	**xxx.x**
Hb g/l	**NUMBER XXX**
WCC number / cumm<br>• Lymphocytes<br>• Neutrophils	**NUMBER XXX.X**
Platelets number/cumm	**NUMBER XXXX**
PT seconds	**NUMBER XX**
APTT seconds	**NUMBER XXX**
INR ratio	**NUMBER X.X**
ALT U/L	**NUMBER XXXX**
Bilirubin µmol/L	**NUMBER XXX.X**
Urea mmol/L	**NUMBER XX.X**
Creatinine µmol/L	**NUMBER XXX.X**
Na mmoll/L	**NUMBER XXX**
K mmoll/L	**NUBMER XX.X**
Cl mmoll/L	**NUMBER XXX**
d-Dimers µg/L	**NUMBER XXXXXX**
Ferritin µg/L	**NUMBER XXXXXX**
CXR signs of lower resp infection	**Yes / No**
Other<br>• specify	**entries with free text**

Daily Organ Support 24 hours (00:00 to 24:00) this is done every day, like the information above

Respiratory Yes/No<br>If 'Yes' t*ick relevant box below to select*	• Non Invasive<br>○ High Flow<br>○ CPAP<br>○ BiPAP<br>• Invasive Mechanical ventilation<br>• High Frequency Oscillation
Cardiovascular<br>Vaso active infusions Yes/No, if Yes ...tick relevant box	• Dopamine<br>• Dobutamine<br>• Adrenaline<br>• Noradrenaline<br>• Vasopressin<br>• Milrinone<br>Other – please specify
Haemofiltration/Haemodialysis Yes/No	
ECMO Yes/No	

Subsequent Research blood samples: date and time – DD:MM:YY HH:MM

To derive duration from time of admission: __:__ (hh:mm) and other events such as death and blood cultures

Time point (tp1 to tp7)	Date and time	Clinical Correlation eg: admission; onset of hypotension, cardiac arrest etc.,

Procedures during admission:

Endotracheal Intubation: Y/N

Central Venous Line Insertion: Y/N

Arterial Line Insertion: Y/N

Chest Drain insertion: Y/N

Date of procedure: DD/MM/YYYY

Other:

Please specify

Surgery during PCCU admission: Yes/No DATE TIME DD:MM:YY HH:MM

If yes, please specify:

DATE AND TIME DD:MM:YY HH:MM

To derive relation to admission to PCCU and research sample date and time

Microbiological investigations (includes bacterial, viral, fungal or any other investigations to identify a causative organism for illness)

1. Blood culture: *Provision to input more than one entry for all categories from 1 to 8*

Requested Y/N

If Y, Result: Positive/Negative

If 'Positive' - please specify

If positive is this significant or no significant? Check box.

Free text for the bacterium

DATE AND TIME DD:MM:YY HH:MM

To derive relation to research sample date and time

2. Naso-pharyngeal aspirate for respiratory panel:

Requested: Yes/No

If yes, result:

Influenza A PCR RNA
Influenza B PCR RNA
RSV PCR RNA
Parainfluenza PCR RNA
Rhinovirus/Enterovirus PCR
HMPV PCR RNA
Adenovirus PCR DNA
Mycoplasma PCR DNA
Enterovirus PCR RNA
Coronavirus PCR
Positive/Negative

If 'Positive' – please specify

DATE AND TIME DD:MM:YY HH:MM

To derive relation to research sample date and time

3. COVID-19 test:

Requested: Yes/No

Throat swap: Yes/No

If Yes- Positive/Negative?

non Bronchoscopic Alveolar Lavage (nBAL): Yes/No

If 'Yes' – Positive/Negative?

DATE AND TIME DD:MM:YY HH:MM

To derive relation to research sample date and time

4. Urine MCS

Requested: Yes/No

If yes, result: Positive/Negative

If 'Positive' – please specify

DATE AND TIME DD:MM:YY HH:MM

To derive relation to research sample date and time

5. CSF MCS

Requested: Yes/No

If yes, result: Positive/Negative

If 'Positive' – please specify

DATE AND TIME DD:MM:YY HH:MM

To derive relation to research sample date and time

6. Non Bronchoscopy Alveolar Lavage (NBAL)

Requested: Yes/No

If yes, result: Positive/Negative

If 'Positive' – please specify

DATE AND TIME DD:MM:YY HH:MM

To derive relation to research sample date and time

7. Sputum culture

Requested: Yes/No

If yes, result: Positive/Negative

If 'Positive' – please specify

DATE AND TIME DD:MM:YY HH:MM

To derive relation to research sample date and time

8. Other specimens/Tests

Please specify

(Prompt to add multiple distinct entries)

DATE AND TIME DD:MM:YY HH:MM

To derive relation to research sample date and time

Medications during PCCU admission:

1. Corticosteroid: Yes/No
   If 'Yes', please tick relevant box/boxes to select
   Prednisolone
   Methylprednisolone
   Hydrocortisone
   Dexamethasone

   Other: *please specify*
   Indication

   Dose (Mg/Kg)

   Date started DD/MM/YY
   Date stopped DD/MM/YY
   To derive duration and relation to date of admission to PCCU

2. Antibiotic: Yes/No
   If 'Yes', please tick relevant box/boxes to select

   Co-Amoxiclavulonic acid (Augmentin)
   Cefotaxime
   Ceftriaxone
   Tazocin
   Meropenem
   Azithromycin
   Clarithyromycin

   Other*: please specify*

   Indication:
   Date started: DD:MM:YY
   Date stopped: DD:MM:YY
   To derive duration and relation to date of admission to PCCU

3. Antiviral Yes/No
   If yes, please tick relevant box/boxes to select

Aciclovir
Gancliclovir

Other: *please specify*

Indication:

Date started: DD/MM/YY
Date stopped: DD/MM/YY
To derive duration and relation to date of admission to PCCU

4. Antifungal Y/N
   If 'Yes', please tick relevant box/boxes to select

   Fluconazole
   Ambisome
   Itraconazole

   Other: please specify

   Indication:

   Date started: DD/MM/YY
   Date stopped: DD/MM/YY
   To derive duration and relation to date of admission to PCCU

5. Anti-inflammatory medications : Yes/No
   If 'Yes', please tick relevant box/boxes to select

   Ibuprofen
   Other NSAID
   Other: please specify

   Indication:

   Date started: DD/MM/YY
   Date stopped: DD/MM/YY
   To derive duration and relation to date of admission to PCCU
6. Other medications modifying immunity :: Yes/No
   (Includes chemotherapy or Antibody based medications)
   If 'Yes', please tick relevant box/boxes to select

   Intravenous Immunoglobulin (IVIG)
   Methotrexate
   Vincristine
   Daunorubicin

Infliximab
Anakinra
Tocilizumab

Other: please specify

Indication:

Date Started: DD/MM/YY

Date Stopped: DD/MM/YY

To derive duration and relation to date of admission to PCCU

Clinical Outcome:

1. Duration on mechanical ventilation: __(DD)
2. Duration on Vase-active agents:__(DD)
3. Date of discharge from PCCU: DD/MM/YY
   To derive duration of stay in PCCU: ___ (DD)
4. Alive at discharge: Yes/No
   If No to 4, (clinical outcome is death), Date of death: DD/MM/YY
   To derive duration of stay in PCCU
   To derive duration from research blood samples
   Withdrawal of life support: Yes/No?
5. Transfer to different hospital: Y/N

Confirmed diagnosis at time of discharge from PCCU: Yes/No

(In case of infectious illness – please specify if microbiologically confirmed)

If 'Yes' please specify

(Please include microbiological diagnosis as well as the complications e.g.: exacerbation of asthma secondary to Rhinovirus infection)

This may involve conclusion following Multi-professinal discussions at the time including Infectious Disease specialist, Microbiologist or any other specialists.

Indeterminate diagnosis at discharge from PCCU: Yes/No

:

Final diagnosis available from clinical follow up Yes/No

If 'Yes' please give details

Is the Adjudicator's diagnosis same as the final diagnosis on follow up?

Yes/No

Clinical Phenotyping

Consensus between two clinicians? Yes /No

If no..

FINAL ADJUDICATOR OPINION:

if yes..

CONSENSUS CLINICAL PHENOTYPING:

Infectious Illness: Yes ☐ No ☐

If infectious illness..

please choose:

- Suspected ☐ (or)
- Proven by positive culture, tissue stain, or PCR test ☐ (or)
- a clinical syndrome associated with a high probability of infection ☐

Type of infectious illness:

Not sepsis ☐

Sepsis ☐

If sepsis..

Choose microbiological agent:

- Sepsis with confirmed bacterial infection ☐
- Sepsis with confirmed other microbiological (eg. Viral, fungal) infection ☐
- Sepsis with no microbiological confirmation ☐

Choose severity of sepsis:

- Sepsis ☐
- Sepsis associated organ dysfunction cardiovascular or non-cardiovascular organ dysfunction ☐
- Septic shock ☐

Diagnosis

Non-infectious illness – please state diagnosis ______________________

Sterile inflammatory illness ☐ eg. Trauma (or) Asthma

Non inflammatory illness ☐ eg. DKA or Status Epilepticus

Infectious illness – please state diagnosis _______________________

Adverse events: Yes/No

If 'Yes', please specify details:

Date and Time: DD/MM/YY

(DATE FOR USE ONLY BY SPONSOR)

Please derive duration from admission to PCCU

Was it related to research?

If yes, please specify if expected from research procedures or unexpected

Was the adverse event considered serious? : Yes/No

If yes, please specify details why it was considered serious:

Has the SAE (related) been reported to REC/Sponsor: Yes/No

(Deaths or complications from acute illness are not considered serious adverse events)
