# Supplementary material for: Immune and metabolic markers for identifying and investigating severe Coronavirus disease and Sepsis in children and young people (pSeP/COVID ChYP study): protocol for a prospective cohort study
Source: BMJ Open. 2023 Mar 27;13(3):e067002. doi: 10.1136/bmjopen-2022-067002 (PMC10069273; doi:10.1136/bmjopen-2022-067002)
Supplement: Supplementary data [file bmjopen-2022-067002supp002.pdf]

Case Report Form (CRF) review tool for clinical phenotyping

Criteria for sepsis

Paediatric consensus conference sepsis criteria, 2005

Presence of Systemic Inflammatory Response Syndrome with suspected or proven infection.

Description of criteria for the PCCS definition of sepsis:

Systemic Inflammatory Response Syndrome (SIRS)

The presence of at least two of the following four criteria, one of which must be abnormal temperature or leukocyte count: This is during any point from first contact with health care professionals or during stay in PCCU.

- Core temperature >38.5C or <36C ☐
- Tachycardia, defined as a mean heart rate of >2SD above normal for age ☐
  Or (for infants, bradycardia < 10th centile for age)
- Mean respiratory rate > 2SD above normal for age or mechanical ventilation for an acute process ☐
- Leucocyte count elevated or depressed for age ☐

Evidence of infection includes positive findings on clinical exam, imaging, laboratory tests (e.g., white blood cells in a normally sterile body fluid, perforated viscus, chest radiograph consistent with pneumonia, petechial or purpuric rash, or purpura fulminans)

Age specific vital signs and laboratory parameters:

Age Group[a]	Heart Rate, Beats/Min[b,c]		Respiratory Rate, Breaths/Min[d]	Leukocyte Count, Leukocytes × 10^3/mm[3b,c]	Systolic Blood Pressure, mm Hg[b,c,e,f]
	Tachycardia	Bradycardia			
0 days to 1 wk	>180	<100	>50	>34	<65
1 wk to 1 mo	>180	<100	>40	>19.5 or <5	<75
1 mo to 1 yr	>180	<90	>34	>17.5 or <5	<100
2–5 yrs	>140	NA	>22	>15.5 or <6	<94
6–12 yrs	>130	NA	>18	>13.5 or <4.5	<105
13 to <18 yrs	>110	NA	>14	>11 or <4.5	<117

Surviving sepsis campaign for sepsis guidelines in children, 2020

- **Defined as per the PCCS 2005 guideline (OR) evidence of *severe bacterial infection leading to life threatening organ dysfunction would be included, even when there is deviation from the specific 2005 criteria above.***

Study ID:

Consensus between two clinicians Yes /No

If 'No'

Final Adjudicator opinion sought Yes/No

Acute illness: Yes ☐ No ☐

Infectious Illness: Yes ☐ No ☐

If infectious illness, please state:

- Suspected ☐ (or)
- Proven by positive culture, tissue stain, or PCR test ☐ (or)
- a clinical syndrome associated with a high probability of infection ☐

Type of infectious illness:

Not sepsis ☐

Sepsis ☐ If sepsis, please choose one below

- Sepsis with confirmed bacterial infection ☐
- Sepsis with confirmed viral infection ☐
- Sepsis with no microbiological confirmation ☐

Severity of sepsis is defined based on organ function in to

- Sepsis ☐
- Sepsis associated organ dysfunction cardiovascular or non-cardiovascular organ dysfunction ☐
- Septic shock – sepsis with cardiovascular organ dysfunction with evidence of hypotension, need for treatment with vasoactive medication or impaired perfusion. ☐

Diagnosis

Non-infectious illness – please state diagnosis ______________________

Sterile inflammatory illness ☐ eg. Trauma (or) Asthma

Non inflammatory illness ☐ eg. Diabetic Ketoacidosis or prolonged febrile seizure

Infectious illness – please state diagnosis _______________________

Comments:
