# Supplementary material for: Immune and metabolic markers for identifying and investigating severe Coronavirus disease and Sepsis in children and young people (pSeP/COVID ChYP study): protocol for a prospective cohort study
Source: BMJ Open. 2023 Mar 27;13(3):e067002. doi: 10.1136/bmjopen-2022-067002 (PMC10069273; doi:10.1136/bmjopen-2022-067002)

Clinical phenotyping of patients recruited to pSeP/COVID study
Decision flow chart for reviewers

v 0.1

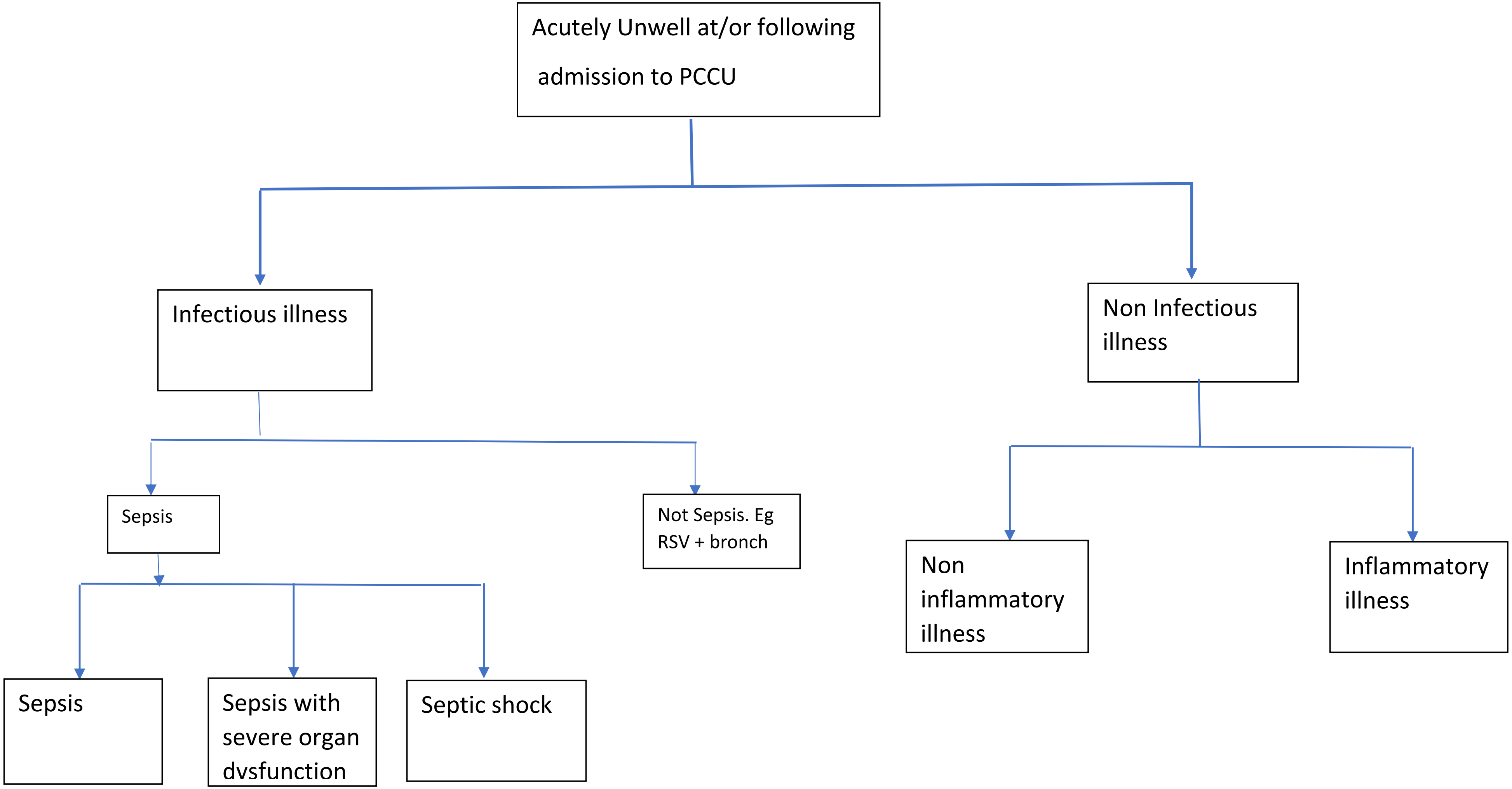

Supplement: Supplementary data [file bmjopen-2022-067002supp003.pdf]
