# Supplementary material for: Immune and metabolic markers for identifying and investigating severe Coronavirus disease and Sepsis in children and young people (pSeP/COVID ChYP study): protocol for a prospective cohort study
Source: BMJ Open. 2023 Mar 27;13(3):e067002. doi: 10.1136/bmjopen-2022-067002 (PMC10069273; doi:10.1136/bmjopen-2022-067002)
Supplement: Author's manuscript [file bmjopen-2022-067002.draft_revisions.pdf]

BMJ Open

BMJ Open is committed to open peer review. As part of this commitment we make the peer review history of every article we publish publicly available.

When an article is published we post the peer reviewers' comments and the authors' responses online. We also post the versions of the paper that were used during peer review. These are the versions that the peer review comments apply to.

The versions of the paper that follow are the versions that were submitted during the peer review process. They are not the versions of record or the final published versions. They should not be cited or distributed as the published version of this manuscript.

BMJ Open is an open access journal and the full, final, typeset and author-corrected version of record of the manuscript is available on our site with no access controls, subscription charges or pay-per-view fees (http://bmjopen.bmj.com).

If you have any questions on BMJ Open's open peer review process please email info.bmjopen@bmj.com

Immune and metabolic markers for identifying and investigating severe Coronavirus disease and Sepsis in children and young people. (pSeP/COVID ChYP study) – Protocol for a prospective cohort study

Journal:	*BMJ Open*
Manuscript ID	bmjopen-2022-067002
Article Type:	Protocol
Date Submitted by the Author:	28-Sep-2022
Complete List of Authors:	Oruganti, Sivakumar; University Hospital of Wales, Paediatric Critical Care Unit<br>Rodrigues, Patrícia R.S.; Cardiff University,<br>White, Daniel; Cardiff University<br>Watkins, W John; Cardiff University, Cochrane Institute of Primary Care and Public Health<br>Shapey , Selyf; Noah's Ark Children's Hospital for Wales, Paediatric Critical Care Unit<br>Barrow, Anna; Noah's Ark Children's Hospital for Wales, Paediatric Critical Care Unit<br>al Samsam, Rim; Noah's Ark Children's Hospital for Wales, Paediatric Critical Care Unit<br>Ali, Sara; Noah's Ark Children's Hospital for Wales, Paediatric Critical Care Unit<br>Gajraj, Malcolm; Noah's Ark Children's Hospital for Wales, Paediatric Critical Care Unit<br>Skone, Richard; Noah's Ark Children's Hospital for Wales, Department of Paediatric Intensive Care<br>Jardine, Michelle; Noah's Ark Children's Hospital for Wales, Paediatric Critical Care Unit<br>Evans, Jennifer; Noah's Ark Children's Hospital for Wales, Paediatric Critical Care Unit<br>Struik, Siske; Noah's Ark Children's Hospital for Wales, Paediatric Critical Care Unit<br>Song, Jong; Noah's Ark Children's Hospital for Wales, Paediatric Critical Care Unit<br>Abood, Lloyd; Morriston Hospital<br>Paquete, Barbara; Noah's Ark Children's Hospital for Wales, Paediatric Critical Care Unit<br>Foulkes, Sian; Noah's Ark Children's Hospital for Wales, Paediatric Critical Care Unit<br>Saunders, Benjamin; Noah's Ark Children's Hospital for Wales, Infectious Diseases services for Wales<br>Strang, Angela; Cardiff University<br>Phillips, Bethan; University Hospital of Wales, Children's and Young Adults Research Unit<br>Evans, Awen; University Hospital of Wales, Children's and Young Adults Research Unit

Buchanan, Iona; University Hospital of Wales, Children's and Young Adults Research Unit
Bowes, Susan; University Hospital of Wales, Children's and Young Adults Research Unit
Ali, Begum; University Hospital of Wales, Children's and Young Adults Research Unit
Gore, Maya; University Hospital of Wales, Children's and Young Adults Research Unit
Thomas-Turner, Rhian; University Hospital of Wales, Children's and Young Adults Research Unit
Andrews, Robert; Cardiff University
Zaher, Summia; University Hospital of Wales, Department of Obstetrics and Gynaecology
Sharma, Simran; Cardiff University, Infection and Immunity; Cardiff and Vale NHS Trust, Women's unit
Chakraborty, Mallinath; University Hospital of Wales,
Parkinson, Edward; Cardiff University; Cardiff University
Liberatore, Federico; Cardiff University
Woolley, Thomas; Cardiff University
Edkins, Sarah; Cardiff University
Davies, Luke C.; Cardiff University
Moet, Linda; Cardiff University,
McLaren, James E.; Cardiff University
Watson, Gareth L.; Cardiff University
O'Donnell, Valerie; Cardiff University College of Biomedical and Life Sciences, Centre for Trials Research
Hood, Kerry; Cardiff University College of Biomedical and Life Sciences, Centre for Trials Research
Ghazal, Peter; Cardiff University, Medical School

Keywords: Paediatric intensive & critical care < ANAESTHETICS, INFECTIOUS DISEASES, COVID-19

SCHOLARONE™ Manuscripts

Immune and metabolic markers for identifying and investigating severe Coronavirus disease and Sepsis in children and young people. (pSeP/COVID ChYP study) – Protocol for a prospective cohort study

Sivakumar Oruganti[1,2], Patrícia R.S. Rodrigues[2], Daniel White[2], William John Watkins[2], Selyf Shapey[1], Anna Barrow[1], Rim al Samsam[1], Sara Ali[1], Malcolm Gajraj[1], Richard Skone[1], Michelle Jardine[1], Jennifer Evans[3], Siske Struik[3], Jong Eun Song[1], Lloyd Abood[4], Barbara Paquete[1], Sian Foulkes[1], Benjamin Saunders[3] Angela Strang[2], Bethan Phillips[5], Awen Evans[5], Iona Buchanan[5], Susan Bowes[5], Begum Ali[5], Maya Gore[5] and Rhian Thomas-Turner[5], Robert Andrews[2,] Summia Zaher[2,6], Simran Sharma[2,6], Mallinath Chakraborty[2,7] Edward Parkinson[8], Federico Liberatore[8], Thomas E Woolley[9], Sarah Edkins[2], Luke C. Davies[2], Linda Moet[2], James E. McLaren[2], Gareth L. Watson[10], Valerie O'Donnell[2], Kerry Hood[10], Peter Ghazal[2]

[1]Regional Paediatric Intensive Care Unit, University Hospital of Wales, Cardiff, UK
[2] School of Medicine, Cardiff University, Cardiff, UK
[3]Infectious Disease Service for Wales, Noah's Ark Children's Hospital, Cardiff, UK
[4]Morriston Hospital, Swansea, Wales, UK
[5]Children's and Young Adults Research Unit, University Hospital of Wales, Cardiff ,UK
[6]Department of Obstetrics and Gynaecology, University Hospital of Wales, Cardiff, UK
[7]Regional Neonatal Intensive Care Unit, University Hospital of Wales, Cardiff, UK
[8]School of Computer Science and Informatics, Cardiff University.
[9] School of Mathematics, Cardiff University, Senghennydd Road, Cardiff, UK
[10]Centre for Trials Research, Cardiff University, Cardiff UK

Corresponding Author

Dr Sivakumar Oruganti
Paediatric Critical Care Unit
Noah's Ark Children's Hospital
University Hospital of Wales
Health Park
Cardiff
CF14 4XW
Phone: 02720 746423
Email: siva.oruganti@wales.nhs.uk

For peer review only

ABSTRACT

Introduction

Early recognition and appropriate management of paediatric sepsis are known to improve outcomes. A previous systems biology investigation of the systemic immune response in neonates to sepsis identified immune and metabolic markers that showed high accuracy for detecting bacterial infection. Further gene expression markers have also been reported previously in the paediatric age group for discriminating sepsis from control cases. More recently, specific gene signatures were identified to discriminate between COVID-19 and its associated inflammatory sequelae. Through the current prospective Cohort study, we aim to evaluate immune and metabolic blood markers which discriminate between sepsis (including COVID-19) from other acute illnesses in critically unwell children and young persons, up to 18 years of age.

Methods and analysis

We describe a prospective cohort study for comparing the immune and metabolic whole blood markers in patients with sepsis, COVID-19 and other illnesses. Clinical phenotyping and blood culture test results will provide a reference standard to evaluate the performance of blood markers from the research sample analysis. Serial sampling of whole blood (50µl each) will be collected from children admitted to intensive care and with an acute illness to follow time dependent changes in biomarkers. An integrated lipidomics and RNASeq transcriptomics analyses will be conducted to evaluate immune-metabolic networks that discriminate sepsis and COVID-19 from other acute illnesses. This study received approval for deferred consent.

Ethics and dissemination

The study has received research ethics committee approval from the Yorkshire and Humber Leeds West Research Ethics Committee 2 (reference 20/YH/0214; IRAS reference 250612). Submission of study results for publication will involve making available all anonymised primary and processed data on public repository sites. This study has been registered in the Clinical Trials database register. (NCT04904523*)*

STRENGTHS AND LIMITATIONS OF THIS STUDY

► Multi-modal investigation of molecular biomarkers for the early diagnosis of paediatric sepsis using a minimally invasive low blood volume.

► Identification of host–response molecular patterns associated with various infections (bacterial, viral and fungal).

► Immunophenotyping, immunoassays, lipidomics and functional analysis will be performed during the hospital journey and in cases of suspected sepsis to investigate time-dependent changes in biomarkers of sepsis.

►Potential selection bias towards younger cases

►Single site observational study

For peer review only

INTRODUCTION

Sepsis is a global health problem affecting all age groups. A systematic review of published literature from 1979 to 2016 was carried out to estimate population-based sepsis incidence in neonates and children. Reports from 15 studies in 12 countries showed an aggregate estimate of 48 (95% CI 27–86) sepsis cases and 22 (14–33) severe sepsis cases in children per 100,000 person-years. The population-level estimate for neonatal sepsis was 2202 (95% CI 1099–4360) per 100,000 livebirths, with mortality between 11% and 19%. Extrapolating on a global scale gave an estimated incidence of approximately 3 million cases of sepsis in neonates and 1.2 million cases in children annually. Mortality ranged from 1% to 5% for sepsis and 9% to 20% for severe sepsis.[1]

Definition of sepsis in children

At present, sepsis is defined in adults as life-threatening organ dysfunction resulting from a dysregulated host response to infection [2]. This definition has removed the terms 'Systematic Inflammatory Response Syndrome' (SIRS) and 'severe sepsis' found in previous definitions and highlights organ dysfunction for clinical management. In children, the existing Surviving Sepsis consensus conference definition of sepsis based on the criteria for SIRS in the presence of confirmed or suspected infection is still used for identification of sepsis and clinical research [3]. The recently updated guidance for the management of sepsis in children (2020) recognises the clinical heterogeneity of this illness in children, accounting for life-threatening organ dysfunction [4]. A revised definition of sepsis by the paediatric sepsis definition task force is awaited [5]. Recently we put forward a setpoint mechanism for understanding sepsis. This suggests that *dysregulation* of the host response to infection in sepsis is explainable by regulatory shifts in homeostasis **setpoints.** This is due to negative and positive feedback changes in the concerted behaviour of multiple (immune, metabolic, and neuronal) systems in response to infection. We hypothesise that infants and children have age-dependent metabolic demands and a developing immune setpoint that increases the risk of sepsis [6]. Sepsis can arise from any type of infection - bacterial, viral, fungal, or parasitic [4]. Even though less common in children, COVID-19 can be associated with severe illness. History of infection and age-dependent changes in the immune system give rise to subtle but important differences in response to SARS-CoV-2 infection in children when compared to adults. In the early life and younger years, the innate response is a dominant feature of the immune response to infection. In the case of SARS-CoV-2 infection, children are known to mount a stronger and more vigorous Interferon (IFN) antiviral response than adults, curtailing viral replication at an early stage of infection[7].

Biomarkers in sepsis

Several host inflammatory biomarkers have been suggested to improve the ability for early recognition of sepsis. These include C-Reactive Protein (CRP), Pro-calcitonin (PCT), Interleukin-8, soluble CD14 (presenilin) and triggering receptor expressed on myeloid cells (sTREM-1) that have all been evaluated without conclusive results [8]. It is important to recognise that sepsis is a complex pathophysiological process comprising an immune response which interacts with the body's metabolic and physiologic functions. A profile of multiple markers capturing the complexity of the pathophysiological response would be better than an individual marker to detect and study the disease process [9]. In neonatal sepsis, it has been shown that the inclusion of metabolic markers as well as adaptive immune responses with innate immune signal changes, increased the accuracy for early recognition of sepsis [10]. These complex molecular changes during sepsis correlate with altered gene expression[11] and

For peer review only

in some infectious illnesses, there was a unique signature alteration in gene expression with a demonstrable value in understanding pathogenicity [12-14]. Gene expression markers have been evaluated to identify bacterial infections in febrile children [15] and differences in transcriptomic profiles in blood have been used to discriminate clinical phenotypes and sub-groups of sepsis [16,17].

Coronavirus disease (COVID-19) in children and young people admitted to paediatric intensive care

Infection with Severe Acute Respiratory Syndrome-Coronavirus 2 (SARS-CoV-2) is mild or asymptomatic in the majority of children [18,19]. The proportion of children presenting to the hospital who have severe COVID-19 and require admission to intensive care has been variable ranging from 1.75% in China [20] to a higher rate of 9.7% to 28% in the western world [21-23]. The clinical risk factors for severe COVID-19 in children have been reported to be age over 10 years, black and Asian ethnic groups, co-morbidities and obesity [23,24]. The difference in the severity of COVID-19 in children, when compared to adults and the elderly, has been studied extensively. Various factors to explain this difference have been reported and include vasculopathy, the density of Angiotensin-Converting Enzyme 2 (ACE 2) receptors, age-based differences in coagulation profiles and immune responses [25], and heightened antiviral IFN response (Yoshida 2021) [7]. Paediatric Inflammatory Multisystem Syndrome Temporally related to SARS-CoV-2 (PIMS-TS) also called Multisystem Inflammatory Syndrome in Children (MIS-C) is a hyperinflammatory syndrome seen in some children following contact with SARS-CoV-2. Two phenotypes have been described – high fevers with severe gastrointestinal symptoms; and shock with myocardial dysfunction and Kawasaki disease-like clinical features. Research to evaluate the pathophysiology of PIMS-TS reported an abnormal immune response mediated by Super Antigen (SAg) activity of the SARS-CoV-2 S glycoprotein [26]. It has also been associated with immune markers related to endothelial dysfunction in children[27]. Through this study, we hope to compare immune responses in children with COVID-19, PIMS-TS and sepsis.

Study methods for molecular and cellular analysis

The studies so far have been restricted to using transcriptomic analysis for an observational case-control methodology to identify differentially expressed genes in children with sepsis (cases) when compared to children without an acute illness (controls). Evaluation of differentially expressed genes in a particular illness, when compared to healthy controls, helps in understanding the biological pathways and pathophysiology of the disease processes. However, the use of healthy controls in studies aiming to identify gene expression markers that discriminate a particular disease from other illnesses may lead to spectrum bias and fail to account for time-resolved differences [28]. Further, the use of other modal data inclusive of metabolites, in particular lipids, proteomic, cellular and clinical observations allow for a more complete picture of the underlying pathophysiology and diagnostic algorithm development. We propose a prospective cohort methodology to investigate the differentially expressed genes over time in children with sepsis and other acute illnesses.

METHODS

Hypothesis and specific aims

A neonatal-sepsis classifier integrating immune and metabolic pathways was able to discriminate neonates with bacterial infections and sepsis with high accuracy [10]. We hypothesise that the integrative tripartite immune-metabolic pathways represented by

For peer review only

differentially expressed genes between sepsis and other illnesses would discriminate bacterial infection causes of sepsis from COVID-19 illness and other acute non-infectious illnesses as well vary between different age groups in children under 18. Our specific aims include:

- Compare immune and metabolic markers which discriminate sepsis and COVID-19 from other acute non-infectious illnesses in children admitted to PICU.
- Investigate the evolution of immune responses in children with sepsis and COVID using serial samples.
- Evaluate the utility of gene expression markers to discriminate heterogeneous sub-groups of sepsis and other critical illnesses with the help of deep clinical phenotyping.

- Understand physiological processes associated with COVID-19 and PIMS-TS and how they differ from bacterial sepsis aetiology.
- Explore age-dependent differences in systemic host-response and determine whether these differences are associated with the risk of disease severity to infection.

We plan to explore the feasibility of objectively defining the balance between immune tolerance and resistance in general, as well as in different paediatric age groups. This is through investigating pathway biology response to sepsis and multi-omic analyses.

Inclusion criteria

Children or young people under 18 years who

1. Are admitted to paediatric critical care unit and
2. Have an acute illness including trauma and
3. Have routine blood tests as part of their clinical care

(or)

Children or young people under 18 years who are admitted to paediatric wards with confirmed COVID-19 illness or PIMS-TS.

Exclusion criteria

1. Admitted to hospital for social reasons without an acute illness.

2. Declined consent by Parents or carers with legal responsibility, or by competent young person

3. Admitted to PCCU electively without an acute illness

4. Direct clinical care team not able to provide research information in a language appropriate for non-English/Welsh speaking participants

Patient/Family journey (please see Figure 1)

Any child or young person with an acute infectious illness or a non-infectious illness such as trauma on admission to PCCU will be eligible for participation in the Study. All children will be screened by the direct clinical care team and research nurse. In those who are eligible, a research blood sample will be collected only if blood sampling is required for routine clinical care. Further blood samples may be collected but only alongside clinical blood samples and at least 24 hours apart during the illness, aiming to collect a final sample during recovery from organ failure. Data collected will include clinical and laboratory information essential for clinical characterisation including clinical profile, the severity of illness and clinical outcomes.

For peer review only

There is no follow-up planned nor repeat blood samples following discharge from the hospital. Anonymous clinical data during the index hospital admission will be collected.

Case definitions and group stratification criteria

We aim to carry out deep clinical phenotyping of all patients recruited for the Study using anonymous data from the Case Report Forms(CRF) (Appendix 1). Two clinicians will independently review the data and allocate a diagnosis to all recruited patients broadly into 'Infectious illness', and 'Non-infectious illness' with further specific categorization into various specific illnesses including COVID-19 and Sepsis (Appendix 2 & 3, Clinical Phenotyping tool and Clinical phenotyping flow chart). In case of any disagreement among the two clinicians, a third clinician will provide a final decision. This forms the reference standard for the classification of illness to aid the interpretation of the research sample analysis results.

The current practice is to isolate all children admitted to the paediatric critical care unit and wards until their SARS-CoV-2 PCR test result is known. Only those cases that meet the clinical phenotype of COVID-19 illness will be categorised as COVID-19[29]. Patients who are coincidentally positive for SARS-CoV-2 but identified with other illnesses on clinical phenotyping, will not be categorised as COVID-19.

As the revisions to the definition of sepsis are pending, clinical phenotyping will be broadly based on the surviving sepsis consensus definition (2005). Patients will be included if they meet 2 or more criteria of Systemic Inflammatory Response Syndrome (SIRS), with at least one criteria related to white cell count or temperature, and with confirmed or strongly suspected to have an infection [3]. There have been algorithms used previously to identify bacterial infections [30,31]. In this Study we plan to evaluate sepsis due to any aetiology including fungal or viral infections. The clinical phenotyping will aid in categorising patients into descriptive clinical clusters which we believe would have more utility in studying differences in immune metabolic biological pathways between sepsis due to viral, bacterial or fungal sepsis or where there is no microbiological agent identified. The process aims to report those cases which may deviate from the 2005 consensus definition, but have strongly suspected or confirmed infection and life threatening organ dysfunction. In addition, we can study the differences in sepsis as a whole in comparison with other acute illnesses.

Recruitment and consent

Recruitment of eligible children admitted to the paediatric critical care unit meets the criteria for emergency research as we need to collect research blood samples soon after admission to the critical care unit along with routine blood tests and it would be inappropriate to approach parents or legal guardians for consent [32]. We have ethics approval for research before consent and collection of multiple samples if the research team find parents not ready to make an informed choice to consent for research. Consultee advice will be sought in patients who are 16 and 17 years old who lack capacity or are not able to make an informed choice due to the critical illness.

In patients admitted to wards with confirmed COVID-19 or PIMS-TS, consent will be obtained after appropriate information sharing before research sample collection at the time of routine clinical blood sampling.

Patient and Public involvement

The study group have engaged with representative members of public for the design of the study. Specific areas where this has led to incorporation of ideas and feedback are listed below:
:

For peer review only

1. Justification for the Study: Early identification of severe infectious illness will provide a diagnosis and information on prognosis will help in counselling and communicating with patients, families and parents.
2. Representatives of parents have approved of the methods involving research before consent as this is considered research in an emergency setting. The feedback stressed the importance of timing in the approach to parents of prospective research participants, to allow information sharing and consenting in such a way it causes minimum distress. We agreed that the timing for the approach should be based on the parent's and patients' ability to understand the research and not based on the duration from the collection of the first sample.
3. In view of the importance of the appropriate timing for informed consent, it was also agreed that we could take further samples before consent. This was only in a situation where the direct clinical care team and research nurses felt that the parents may be emotionally not ready for the approach or the prospective research participant was severely unwell.
4. Representative members of the public were involved in drafting the information sheets for parents, carers and older children. The final drafts were approved as appropriate for sharing information with parents and patients at the time of initial approach and consenting. Even though feedback about the information provided was incorporated, the information could not be shortened due to regulatory requirements.
5. Feedback from patient groups through Wales Gene Park included the research methods and information shared with families and legal guardians. Also included specific information related to Next Generation Sequencing in the Study. The feedback was supportive of the methods used and suggestions were incorporated into the Information sheets.

Peer review and feedback were sought through the UK Paediatric Intensive Care Society – Study Group as well as during a previous grant application.

SAMPLE PROCESSING AND DATA ANALYSIS

Sample storage and analysis (please see Figure 1)

The blood samples, at clinical sites, will be stored at –20°C until transferred to the laboratory for processing, where they are stored at -80°C, once consent is obtained.

Blood samples transferred to the laboratory will be processed in batches and initially retained by the pSeP team onsite at Cardiff University in a dedicated lock-secured freezer. For long-term storage and access for future research, samples will be housed by the Cardiff Biobank. For the transcriptomic and lipidomic analysis, the blood is mixed with the stabilising reagent potassium amyl xanthate (PAX) in the collection tubes, all cells are immediately lysed and are not considered to be human tissue. The second tube will be stored as an EDTA-whole blood sample (with or without a stabiliser for cellular phenotyping) and after cell, and targeted proteomics and metabolomics analyses will be banked for future use, for validating future diagnostic platforms.

We aim to apply a systems biology multi-omic analysis of blood samples. This will involve using microarray and RNAseq methodology for probing the transcriptome of whole blood. While for specific immune cell characterisation, we will use techniques such as the Cellular Indexing of Transcriptomes and Epitopes by Sequencing (CITE-Seq) methods that perform RNAseq along with quantitative and qualitative information on surface proteins at a single-cell

For peer review only

level. For metabolite and proteomic analyses, we will use the methods of liquid chromatography with tandem mass spectrometry (LC-MS/MS) in a targeted high-throughput manner, and we will use quantitative lipidomic profiling (LC-MS/MS) for comprehensive screening of specific pathways such as the complement system.

Clinical data

Case Report Forms will be used for collecting anonymous clinical data and also containing the clinical classifier (Figure1) from clinical phenotyping (Appendix 1)

Sample size calculation for transcriptomic analysis using RNAseq

We aim to recruit 250 patients with acute illnesses admitted to our paediatric intensive care unit. Extrapolating the results of previous reports on the prevalence of severe sepsis in the paediatric intensive care population, we estimate that there will be around 25 patients with sepsis included in our study [33,34]. For calculating the power and Type 1 error (α), we refer to the Negative Binomial model for biological variability estimations in human samples used by Hart et al (2013) [35]. Using an RNAseq minimum read depth of 30 million reads per sample and a liberal coefficient of variability (σ) of 0.6, we calculate that to achieve a Power(β) of 0.8 and Type 1 error (α) of 0.01, we would need around 20 samples in each group for a two-fold change in differentially expressed genes.

Data processing and analysis

Paired-end reads from RNAseq Illumina sequencing will be trimmed and processed for quality control [36,37], before mapping to the human genome using Spliced Transcript Alignment to a Reference (STAR) [38]. Once we have read counts per gene/trancript, we plan to use R Language and Environment for Statistical Computing [39] followed by quality control and data processing using Bioconductor packages [40]. A per-gene hypothesis of differential average expression will be tested using a negative binomial generalised linear model—DESeq2 package and resulting p-values will be controlled for multiple testing using the Benjamini-Hochberg method [41]. For classification, a variety of machine learning and statistical pathway biology approaches, as described in Smith et al. 2014 [42], will be used. Pathway analyses will be carried out step-wise using a pathway biology approach, becoming more focused. For metabolomic and proteomic data, absolute concentrations (determined by liquid chromatography Mass Spectrometry LC-MS/MS) of analytes in extracts from blood samples will apply validated tools such as MetaboAnalyst 4.0 [43]. Further multivariate statistical testing (PCA, PLSDA, Random Forest, ANOVA) using group assignment derived from clinical phenotyping (types of sepsis and between sepsis and non-septic controls), will also be used to determine metabolites or proteins that are significantly different in abundance between clinical phenotypes. Single cell analyses (CITE-seq) using surface protein and RNA libraries (10x Genomics) and next-generation sequencing will be multiplexed by cell "hashing" [44]. Demultiplexed sequencing data will be aligned to the reference transcriptome using CellRanger (10x Genomics) and the number of unique molecular identifiers (UMI) per cell will be quantified. Computational analyses and quality control will be performed using R packages including Seurat to integrate hashtag, protein and RNA libraries whilst also enabling demultiplexing of donors, doublet detection and cell clustering [45].
For the final pathway-biomarker assessment of the predictive success of the model, Receiver Operator Characteristics (ROC) will be applied.

For peer review only

DISCUSSION

Through this a prospective observational cohort study, we plan to evaluate immune metabolic biological pathways in children with acute illnesses admitted for paediatric critical care. The aim is to evaluate the differences in immune and metabolic pathways in children with sepsis and other illnesses including COVID-19 in children and young people. The use of the cohort design will help in recruiting children of different age groups from birth to under 18 years admitted to paediatric critical care unit with acute illnesses. The subsequent nested case-control analysis of different clinical groups from the cohort matched for age will help to identify differentially expressed genes among the different clinical phenotypes such as COVID-19 and confirmed sepsis with microbiological confirmation, COVID-19 and confirmed other viral infections, PIMS-TS and confirmed viral infection, PIMS-TS and sepsis with confirmed microbiological confirmation, sepsis with microbiological confirmation and confirmed viral infection. The initial analysis in a discovery cohort will be followed by cross-validation in a subsequent validation cohort. We plan to include children with polytrauma as this is considered an example of sterile inflammation.

DISCLOSURE OF INTEREST

All the authors declare there is no conflict of interest.

FUNDING

The study has received funding from the Welsh Government-EU ERDF for Ser Cymru II programme grant (East Wales ERDF Programme grant number 80762-CU-106) for Project Sepsis (PG).

ETHICS APPROVAL AND DISSEMINATION

Ethical approval was obtained from the Wales Research Ethics Committee 2 (IRAS project ID250612, REC ref: 20/YH/0214). Operational approval was received from Health and Care Research Wales and local resource and capacity were supported by the Research and Development Department of the Cardiff and Vale University Health Board. The study is sponsored by Cardiff University. The sponsor or funder does not have any role in the study design data collection or analysis. Submission of study results for publication will involve making available all anonymised primary and processed data on public repository sites. All study results will be presented at national and international conferences and published in peer-reviewed open-access journals.

Author contributors

SO (Principal Investigator): conceptualized the study and contributed to design, engagement with representative members of public, sample size calculation, recruitment of patients, data acquisition, analysis, interpretation of results, writing and editing the manuscript;
PRSR: Investigation, Methodology, Visualization;

For peer review only

DW: Investigation, Methodology;
WJW contributed to sample size considerations and data analysis;
SS[1], AB, RA, SA, MG, RS, MJ, JE, SS[2], JES, LA, BP, SF and BS are involved in the recruitment of patients, data acquisition, clinical phenotyping, analysis and writing of the manuscript;
AS: Ethics, Resources, Data Curation, Project administration (Project Manager);
BP, AE, IB, SB, BA, MG and RT-T contributed to drafting data collection forms, sample storage methods, patient recruitment, data collection and governance.
RA: Formal analysis;
SZ and SS: Contributed to patient information and consenting;
MC: Contributed to considerations around Methodology, Consenting, Sample collection procedures and editing the manuscript;
EP and FL: Quality Assurance, Quality Control and Machine Learning analyses;
TEW: Mathematical modelling and testing setpoint hypothesis;
LCD: Investigation, Methodology;
SE: Resources, Investigation, Project administration, Methodology;
LM: Investigation, Methodology;
JEM: Investigation, Methodology;
GLW and KH: Electronic Case Report Form and Data Management;
VO: Methodology, Resources.
PG (Chief Investigator) contributed to conceptualization, design, engagement with representative members of public, sample analysis methods, data analysis, interpretation of results, writing and editing of the manuscript.

Non author contributors

Helen Thomas, Benjamin Beach and Sarra Beach (representing parents and members of public) contributed to consent methods and information for patients, families and parents.
Representative members of public in Project Sepsis and Wales Gene Park contributed feedback on consent methods and information for patients, families and parents;
Prof Mark Peters (Consultant Paediatric Intensivist, Great Ormond Street Hospital) : Through the UK Paediatric Intensive Care Society Study Group, Prof Peters provided feedback on Study design.

For peer review only

REFERENCES

1. Fleischmann-Struzek C, Goldfarb DM, Schlattmann P, Schlapbach LJ, et al. The global burden of paediatric and neonatal sepsis: a systematic review. Lancet Respir Med. 2018 Mar;6(3):223-230. doi: 10.1016/S2213-2600(18)30063-8.

2. Singer M, Deutschman CS, Seymour CW, et al. The Third International Consensus Definitions for Sepsis and Septic Shock (Sepsis-3). JAMA. 2016;315(8):801-810. doi:10.1001/jama.2016.0287

3. Goldstein B, Giroir B, Randolph, A; International pediatric sepsis consensus conference: definitions for sepsis and organ dysfunction in pediatrics. Pediatr Crit Care Med 2005;6(1):2-8. doi: 10.1097/01.PCC.0000149131.72248.E6.

. Weiss SL, Peters MJ, Alhazzani W, et al. Surviving Sepsis Campaign International Guidelines for the Management of Septic Shock and Sepsis-Associated Organ Dysfunction in Children. Pediatr Crit Care Med. 2020 Feb;21(2):e52-e106. doi: 10.1097/PCC.0000000000002198.

5. Menon K, Schlapbach LJ, Akech S, et al. Pediatric Sepsis Definition-A Systematic Review Protocol by the Pediatric Sepsis Definition Taskforce. Crit Care Explor. 2020 Jun 11;2(6):e0123. doi: 10.1097/CCE.0000000000000123.

6. Peter Ghazal, Patricia R.S. Rodrigues, Mallinath Chakraborty, Siva Oruganti, Thomas E. Woolley, Challenging molecular dogmas in human sepsis using mathematical reasoning, eBioMedicine, Volume 80, 2022. https://doi.org/10.1016/j.ebiom.2022.104031.

7. Yoshida, M., Worlock, K.B., Huang, N. et al. Local and systemic responses to SARS-CoV-2 infection in children and adults. Nature (2021). https://doi.org/10.1038/s41586-021-04345-x

8. Marshall JC, Vincent JL, Fink MP, Cook DJ, Rubenfield G, Foster D et al. Measures, markers, and mediators: Towards a staging system for clinical sepsis. Crit Care Med 2003; 31:1560 –1567. DOI:10.1097/01. CCM. 00000065186.67848.3A

9. Marshall JC, Reinhardt K for the International Sepsis Forum. Biomarkers in Sepsis. Crit Care Med 2005;3(7):2290-98. DOI: 10.1097/CCM.0b013e3181a02afc.

10. . Smith CL, Dickinson P, Forster T, Craigon M, Ross A, Khondoker MR et al. Identification of a human neonatal immune-metabolic network associated with bacterial infection. Nature Communications. 2014;5:4649. DOI: 10.1038/NCOMMS5649.

11. Prucha M, Ruryk A, Boriss H, Moller E, Zazula R, Herold I et al. Expression profiling: toward an application in sepsis Studies. Shock 2004;22(1);29–33. DOI: PMID: 15201698.

12. Ramilo O, Allman W, Chung W, Mejias A, Ardura M, Glaser C. et al. Gene expression patterns in blood leukocytes discriminate patients with acute infections. Blood 2007; 109(5): 2066–2077. DOI:10.1182/blood-2006-02-002477.

13. Fjaerli H O, Bukholm G, Krog A, Skjaeret C, Holden M, Nakstad B. Whole blood gene expression in infants with respiratory syncytial virus bronchiolitis. BMC Infect. Dis 2006;6:175. DOI: 10.1186/1471-2334-6-175.

14. Ardura M I, Banchereau R, Mejias A, Di Pucchio T, Glaser C, Allantaz et al. Enhanced monocyte response and decreased central memory T cells in children with invasive Staphylococcus aureus infections. PLoS ONE 2009; 4(5), e5446. DOI: 10.1371journal.pone..0005446.

15. Herberg JA, Kaforou M, Wright VJ, et al; IRIS Consortium. Diagnostic Test Accuracy of a 2-Transcript Host RNA Signature for Discriminating Bacterial vs Viral Infection in Febrile Children. JAMA. 2016 Aug 23-30;316(8):835-45. doi: 10.1001/jama.2016.11236.

For peer review only

16. Sweeney TE, Azad TD, Donato M, et al. Unsupervised Analysis of Transcriptomics in Bacterial Sepsis Across Multiple Datasets Reveals Three Robust Clusters. Crit Care Med. 2018 Jun;46(6):915-925. doi: 10.1097/CCM.0000000000003084.

17. Wong HR, Cvijanovich NZ, Allen GL, et al. Validation of a gene expression-based subclassification strategy for pediatric septic shock. Crit Care Med. 2011 Nov;39(11):2511-7. doi: 10.1097/CCM.0b013e3182257675.

18. Li B, Zhang S, Zhang R, Chen X, Wang Y, Zhu C. Epidemiological and Clinical Characteristics of COVID-19 in Children: A Systematic Review and Meta-Analysis. Front Pediatr. 2020 Nov 2;8:591132. doi: 10.3389/fped.2020.591132.

19. Otto WR, Geoghegan S, Posch LC, et al. The Epidemiology of Severe Acute Respiratory Syndrome Coronavirus 2 in a Pediatric Healthcare Network in the United States. J Pediatric Infect Dis Soc. 2020 Nov 10;9(5):523-529. doi: 10.1093/jpids/piaa074.

20. Lu X, Zhang L, Du H, et al; Chinese Pediatric Novel Coronavirus Study Team. SARS-CoV-2 Infection in Children. N Engl J Med. 2020 Apr 23;382(17):1663-1665. doi: 10.1056/NEJMc2005073.

21. Tagarro A, Epalza C, Santos M, Sanz-Santaeufemia FJ, Otheo E, Moraleda C, Calvo C. Screening and Severity of Coronavirus Disease 2019 (COVID-19) in Children in Madrid, Spain. JAMA Pediatr. 2020 Apr 8:e201346. doi: 10.1001/jamapediatrics.2020.1346.

22. Verma S, Lumba R, Dapul HM, et al. Characteristics of Hospitalized Children With SARS-CoV-2 in the New York City Metropolitan Area. Hosp Pediatr. 2021 Jan;11(1):71-78. doi: 10.1542/hpeds.2020-001917.

23. Ouldali N, Yang DD, Madhi F, et al,; investigator group of the PANDOR study. Factors Associated With Severe SARS-CoV-2 Infection. Pediatrics. 2021 Mar;147(3):e2020023432. doi: 10.1542/peds.2020-023432.

24. Shekerdemian LS, Mahmood NR, Wolfe KK, et al; International COVID-19 PICU Collaborative. Characteristics and Outcomes of Children With Coronavirus Disease 2019 (COVID-19) Infection Admitted to US and Canadian Pediatric Intensive Care Units. JAMA Pediatr. 2020 Sep 1;174(9):868-873. doi: 10.1001/jamapediatrics.2020.1948.

25. Zimmermann P, Curtis N. Why is COVID-19 less severe in children? A review of the proposed mechanisms underlying the age-related difference in severity of SARS-CoV-2 infections Arch Dis Child 2021;106:429–439. . doi:10.1136/archdischild-2020-320338

26. Rivas MN, Porritt RA, Cheng MH, et al. COVID-19 Associated Multisystem Inflammatory Syndrome in Children (MIS-C): a novel disease that mimics Toxic Shock Syndrome. The superantigen hypothesis, Journal of Allergy and Clinical Immunology (2020), doi: 10.1016/j.jaci.2020.10.008.

27. Borgel D, Chacron R, Grimaud M, et al. Endothelial Dysfunction as a Component of Severe Acute Respiratory Syndrome Coronavirus 2–Related Multisystem Inflammatory Syndrome in Children With Shock. Crit Care Med. 2021 Nov 1;49(11):e1151-e1156. doi: 10.1097/CCM.0000000000005093.

28. Lijmer JG, Mol BW, Heisterkamp S, et al. Empirical evidence of design-related bias in studies of diagnostic tests. JAMA. 1999 Sep 15;282(11):1061-6. doi: 10.1001/jama.282.11.1061. Erratum in: JAMA 2000 Apr 19;283(15):1963. PMID: 10493205.

29. Docherty AB, Harrison EM, Green CA, et al; ISARIC4C investigators. Features of 20 133 UK patients in hospital with covid-19 using the ISARIC WHO Clinical Characterisation Protocol: prospective observational cohort study. BMJ. 2020 May 22;369:m1985. doi: 10.1136/bmj.m1985.

30. Downes KJ, Weiss SL, Gerber JS, et al; Centers for Disease Control and Prevention Epicenters Program. A Pragmatic Biomarker-Driven Algorithm to Guide Antibiotic Use in the Pediatric Intensive Care Unit: The Optimizing Antibiotic Strategies in Sepsis (OASIS) Study. J Pediatric Infect Dis Soc. 2017 Jun 1;6(2):134-141. doi: 10.1093/jpids/piw023.

31. Herberg JA, Kaforou M, Wright VJ, et al; IRIS Consortium. Diagnostic Test Accuracy of a 2-Transcript Host RNA Signature for Discriminating Bacterial vs Viral Infection in Febrile Children. JAMA. 2016 Aug 23-30;316(8):835-45. doi: 10.1001/jama.2016.11236. Erratum in: JAMA. 2017 Feb 7;317(5):538.

32. Health Research Authority. Planning and Improving Research-Policies, standards and legislation: Research in emergency settings. https://www.hra.nhs.uk/planning-and-improving-research/policies-standards-legislation/research-emergency-settings/ (Date accessed: 05/12/2021).

33. Ruth A, McCracken CE, Fortenberry JD, et al. Pediatric severe sepsis: current trends and outcomes from the Pediatric Health Information Systems database. Pediatr Crit Care Med. 2014 Nov;15(9):828-38. doi: 10.1097/PCC.0000000000000254. PMID: 25226500.

34. Weiss SL, Fitzgerald JC, Pappachan J, et al; Sepsis Prevalence, Outcomes, and Therapies (SPROUT) Study Investigators and Pediatric Acute Lung Injury and Sepsis Investigators (PALISI) Network. Global epidemiology of pediatric severe sepsis: the sepsis prevalence, outcomes, and therapies study. Am J Respir Crit Care Med. 2015 May 15;191(10):1147-57. doi: 10.1164/rccm.201412-2323OC. Erratum in: Am J Respir Crit Care Med. 2016 Jan 15;193(2):223-4. PMID: 25734408

35. Hart SN, Therneau TM, Zhang Y, Poland GA, Kocher JP. Calculating sample size estimates for RNA sequencing data. J Comput Biol. 2013 Dec;20(12):970-8. doi: 10.1089/cmb.2012.0283. Epub 2013 Aug 20. PMID: 23961961.

36. Trim Galore: A wrapping tool around Cutadapt and FastQC to consistently apply quality and adapter trimming to FastQ files, with some extra functionality for Mspl-digested RRBS-type libraries. (https://www.bioinformatics.babraham.ac.uk/projects/trim_galore/)

37.FastQC: A quality control tool for high throughput sequence data. Available at https://www.bioinformatics.babraham.ac.uk/projects/fastqc/

38. STAR: ultrafast universal RNA-seq aligner, Alexander Dobin Carrie A. Davis Felix Schlesinger Jorg DrenkowChris Zaleski Sonali Jha Philippe Batut Mark ChaissonThomas R. Gingeras, Bioinformatics, Volume 29, Issue 1, 1 January 2013, Pages 15–21,

For peer review only

39. R Core Team. *R: A Language and Environment for Statistical Computing*. R Foundation for Statistical Computing (2016). Available from http:// www.R-project.org.

40. Huber W, Carey VJ, Gentleman R, et al. Orchestrating high-throughput genomic analysis with Bioconductor. Nat Methods 2015;12(2):115-21. doi: 10.1038/nmeth.3252

41. Benjamini Y, Hochberg Y Controlling the false discovery rate: a practical and powerful approach to multiple hypothesis testing. J R Stat Soc B. 1995;57:289–300

42. Smith CL, Dickinson P, Forster T, et al. Identification of a human neonatal immune-metabolic network associated with bacterial infection. Nat Commun 2014;5:4649. doi: 10.1038/ncomms5649

43. Chong J, Wishart DS, Xia J. Using MetaboAnalyst 4.0 for Comprehensive and Integrative Metabolomics Data Analysis. Current Protocols in Bioinformatics 2019;68(1):e86. doi: 10.1002/cpbi.86.

44. Stoeckius M, Zheng S, Houck-Loomis B, et al. Cell Hashing with barcoded antibodies enables multiplexing and doublet detection for single cell genomics. *Genome Biology* 2018;19(1):224. doi: 10.1186/s13059-018-1603-1

45. Butler A, Hoffman P, Smibert P, et al. Integrating single-cell transcriptomic data across different conditions, technologies, and species. *Nature Biotechnology* 2018;36(5):411-20. doi: 10.1038/nbt.4096

Figure legend

Figure 1: Patient recruitment and Blood Sample analysis workflows

For peer review only

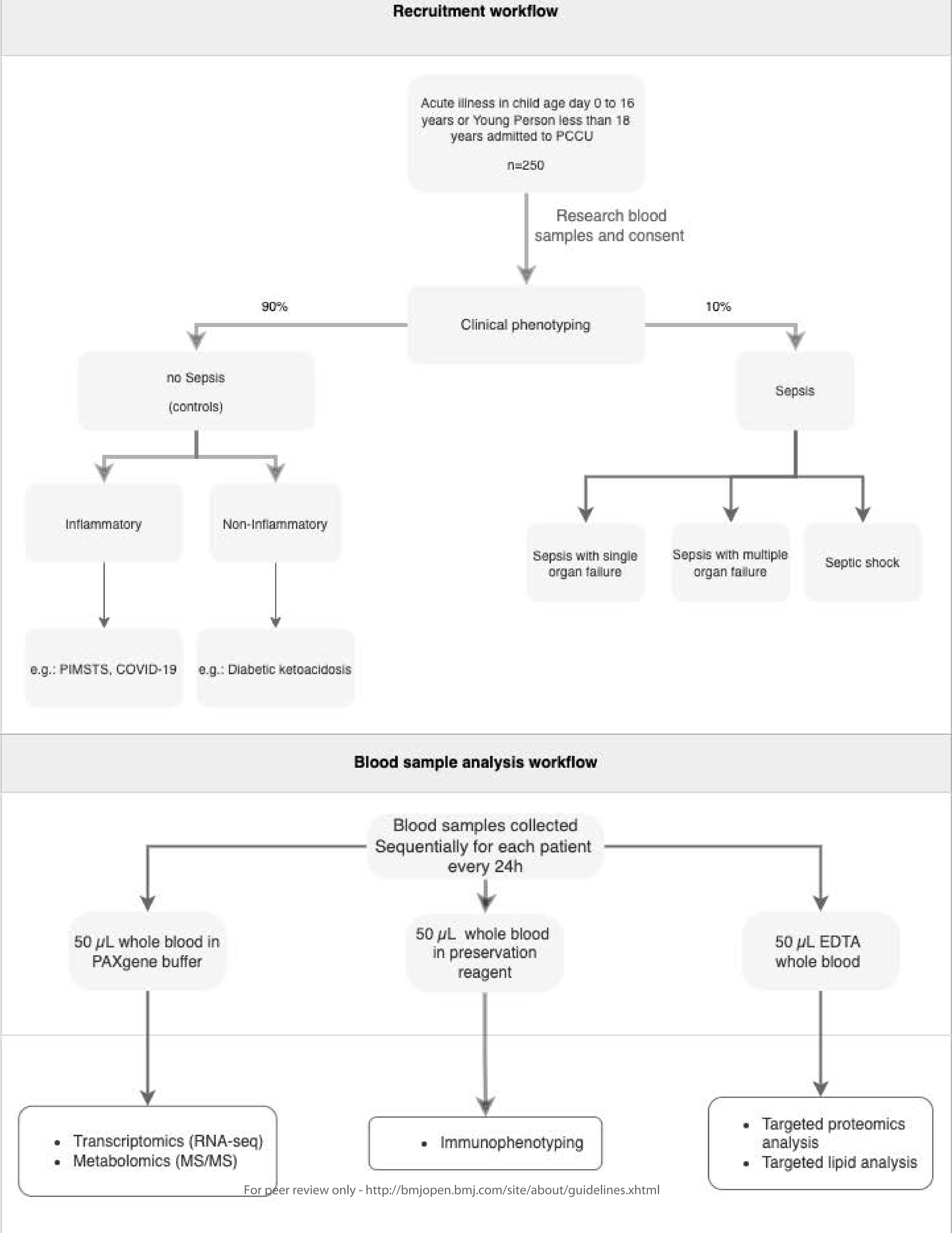
Recruitment workflow
Acute illness in child age day 0 to 16 years or Young Person less than 18 years admitted to PCCU
n=250
Research blood samples and consent
Clinical phenotyping
90%
10%
no Sepsis
(controls)
Sepsis
Inflammatory
Non-Inflammatory
Sepsis with single organ failure
Sepsis with multiple organ failure
Septic shock
e.g.: PIMSTS, COVID-19
e.g.: Diabetic ketoacidosis
Blood sample analysis workflow
Blood samples collected Sequentially for each patient every 24h
Samples collected
50 μL whole blood in PAXgene buffer
50 μL whole blood in preservation reagent
50 μL EDTA whole blood
Analysis
Transcriptomics (RNA-seq)
Metabolomics (MS/MS)
Immunophenotyping
Targeted proteomics analysis
Targeted lipid analysis

For each patient, free text field 300-400 words.

Once data are input, can be reedited in line with new diagnosis.

Research ID number					
Age (in years and months)	*To encode formula to calculate age upon input of date of birth (for confidentiality)*				
Age	<1	2-5	6-12	13-15	16-17
Sex	M	F	Indeterminate		
Ethnicity	White	Black	West Asian		
	East Asian	South Asian			
	Other – please specify				
Weight (kgs)					
Weight centile	< 0.4<br>0.4 to 2<br>2 to 9<br>9 to 75<br>75 to 91<br>91 to 99.6<br>>99.6				

For peer review only

Inclusion Criteria

Admitted to PCCU<br>(Or)<br>Admitted to ward with confirmed COVID positive and/or PIMS-Ts	Yes/No
Acute illness including trauma	Yes/No
Routine blood tests	Yes/No
Age <18 years	Yes/No

Exclusion Criteria

Planned admission to PCCU with no acute illness	Yes/No
Declined Consent for research<br>(Or)	Yes/No

Did not respond to follow up letter	
Language barrier cannot understand English/Welsh	Yes/No

Consent: Yes/No

(Please obtain consent before recording further details)

Co-morbidities: *If yes, please specify or give details regarding co-morbidity (prompt in eCRF)*

Congenital Heart disease Yes / No	Chronic Lung disease Yes/No	Neurological Problem Yes/No
Chronic renal disease Yes/No	Gastrointestinal Yes/No	Asthma Yes/No
Diabetes Yes/No	Liver disease Yes/No	Malignancy Yes/No
Exprematurity Yes/No	Haematological problem Yes/No	Immune disorder (including auto-immune and immune deficiency) Yes/No
Other: please specify		

History available for any vaccination in previous 4 weeks: Yes/No

If 'Yes', what vaccination? :

Acute illness onset timeline

Date of onset of symptoms	DD:MM:YY
Date of admission to hospital	DD:MM:YY
Date of admission to ward	DD:MM:YY
Date of admission to PCCU	DD:MM:YY

Please derive relation to research sample and other info such as microbiological tests and outcome variables

Brief history of illness and background

(*please* ***do not*** *include any patient identifiable information such as name, date of birth, age or address***)**

Admission signs and symptoms

Cough Yes/No	Respiratory distress Yes/No	Wheeze Yes/No	Apnoea Yes/No

For peer review only

Runny nose /Rhinorrhoea Yes/No	Sore throat Yes/No	Recessions Yes/No	Lower chest in drawing Yes/No
Grunting Yes/No	Head Bobbing Yes/No	Colour change Yes/No<br>• Specify:	Cardio Respiratory arrest Yes/No
Palpitations Yes/No	Arrhythmia Yes/no	Cool peripheries Yes/No	Thread /weak peripheral pulses Yes/No
Fever Yes/No	Chills/Rigors Yes/No	Seizure Yes/No	Abnormal movement Yes/No
Lethargy/weakness Yes/No	Headache Yes/No	Photophobia Yes/No	Neck stiffness Yes/No
Focal Neurology Yes/No	Poor tone Yes/No	Pupillary abnormalities Yes/No	Altered Consciousness Yes/No<br>• Irritable<br>• Agitation<br>• Abnormal behaviour<br>• Drowsy Yes/No
Vomiting Yes/No<br>• Bilious Vomiting Yes/No<br>• Bloody Vomiting Yes/No	Diarrhoea Yes/No	Bloody stools Yes/No	Constipation Yes/No
Abdominal distension Yes/No	Abdominal pain Yes/No	Reduced feeds Yes/No	Loss of appetite Yes/No
Weight Loss Yes/No	Skin Rash Yes/No<br>• Maculo/popular Yes/No<br>• Petechial/Ecchymosis Yes/No<br>• Vesicular Yes/No<br>• Other - specify	Lymphadenopathy Yes/No	Bleeding Yes/No
Reduced Urine Output Yes/No	Excessive Urination Yes/no	Haematuria Yes/No	Other Please specify

Admitted following unplanned/emergency surgery: Yes/No

For peer review only

If yes: please specify

PCCU Admission vital observations and laboratory details

FiO2(0.21 -1)	**Number 0.XX**
SpO2 %	**Number XXX**
HR beats/min	**NUMBER XXX**
RR Breaths/min	**NUMBER XXX**
BP mm of Hg<br>• Systolic<br>• Diastolic<br>• Mean	**NUMBER XXX**
Temp Centigrade<br>• Core<br>• Peripheral	**NUMBER XX.X**
GCS	**NUMBER XX**
Blood gas<br>• pH<br>• PaO2 KPa<br>• PaCO2 KPa<br>• Base Excess<br>• Bicarbonate mmol/L	**pH NUMBER X.XX**<br>**PaO2 NUMBER XX.XX**<br>**PaCO2 NUMBER XX.XX**<br>**BASE EXCESS NUMBER XX.XX**<br>**BICARBONATE NUMBER XX.X**
Urine output MLS/KG/HR	**NUMBER XX.XX**
Lactate mmol/L	**NUMBER XX.XX**
CRP mg/L	**NUMBER XXX.X**
Beta D Glucan pg/ml	**XXX.X**
Hb g/l	**XX.X**
WCC number / cumm<br>• Lymphocytes<br>• Neutrophils	**NUMBER XXX.X**
Platelets number/cumm	**NUMBER XXXX**
PT seconds	**NUMBER XX**
APTT second	**NUMBER XXX**
INR ratio	**NUMBER X.X**
ALT U/L	**NUMBER XXXXXX**
Bilirubin µmol/L	**NUMBER XX.X**
Urea mmol/L	**NUMBER XX.X**
Creatinine µmol/L	**NUMBER XXX.X**
Na mmol/L	**NUMBER XXX**
K mmol/L	**NUMBER XX.X**
Cl mmol/L	**NUMBER XXX.X**
d-Dimers µg/L	
Ferritin µg/L	**NUMBER XXXX**
CXR Signs of Lower Resp Infection	**Yes/No**
Other<br>• specify	

For peer review only

First observations since onset of acute illness on Ward / presentation to ED (please circle first if Ward or ED) from local or referring hospital.

FiO2(0.21 - 1	**Number 0.XX**
SpO2 %	**Number XXX**
HR beats/min	**NUMBER XXX**
RR Breaths/min	**NUMBER XXX**
BP mm of Hg<br>• Systolic<br>• Diastolic<br>• Mean	**NUMBER XXX**
Temp Centigrade<br>• Core<br>• Peripheral	**NUMBER XX.X**
GCS	**NUMBER XX**
Blood gas<br>• pH<br>• PaO2 KPa<br>• PaCO2 KPa<br>• Base Excess<br>• Bicarbonate mmol/L	**pH NUMBER X.XX**<br>**PaO2 NUMBER XX.XX**<br>**PaCO2 NUMBER XX.XX**<br>**BASE EXCESS NUMBER XX.XX**<br>**BICARBONATE NUMBER XX.X**
Urine output MLS/KG/HR	**NUMBER XX.XX**
Lactate mmol/L	**NUMBER XX.XX**
CRP mg/L	**NUMBER XXX.X**
Beta D Glucanpg/ml	**XXX.X**
Hb g/l	**XX.X**
WCC number / cumm<br>• Lymphocytes<br>• Neutrophils	**NUMBER XXX.X**
Platelets number/cumm	**NUMBER XXXX**
PT seconds	**NUMBER XX**
APTT second	**NUMBER XXX**
INR ratio	**NUMBER X.X**
ALT U/L	**NUMBER XXXXXX**
Bilirubin μmol/L	**NUMBER XX.X**
Urea mmol/L	**NUMBER XX.X**
Creatinine μmol/L	**NUMBER XXX.X**
Na mmol/L	**NUMBER XXX**
K mmol/L	**NUMBER XX.X**
Cl mmol/L	**NUMBER XXX.X**
d-Dimers μg/L	
Ferritin μg/L	**NUMBER XXXX**
CXR Signs of Lower Resp Infection	**Yes / No**
Other<br>• specify	

For peer review only

Paediatric Index of Mortality (PIM-3) score: decimal. 0-1 (range)

Provisional Diagnosis

1. Provisional Diagnosis	**Comments multiple**
2. Provisional Diagnosis	

Organ Support in the first 24 hours

Respiratory Yes/No<br>If 'Yes' tick relevant box to select	• Non Invasive<br>○ High Flow<br>○ CPAP<br>○ BiPAP<br>• Invasive Mechanical ventilation<br>• High Frequency Oscillation	
Cardiovascular<br>Vaso active infusions Yes/No. If 'Yes' ...tick relevant box to select	• Dopamine<br>• Dobutamine<br>• Adrenaline<br>• Noradrenaline<br>• Vasopressin<br>• Milrinone<br>Other – please specify	
Haemofiltration/Haemodialysis Yes/No		
Extra corporeal life support Yes/No		
Worse PELOD score during illness		

1ST Research blood sample at admission

Date / Time to be entered on CRF: DD:MM:YYYY HH:MM

Suspected sepsis /suspected COVID/ non-septic illness

For peer review only

Daily vital observations and laboratory results (worst and best) - please can you make it easy for Awen and Iona to input data as this is data collected for every day of PICU admission of child?

Date of PCCU admission: _ _DD:MM:YY.

To derive relation to time of admission to PCCU, research sample, adverse events and microbiological sample results

FiO2(0.21 -1.0)<br>• highest<br>• lowest	**NUMBER .XX**
SpO2 %<br>• lowest<br>• highest	**NUMBER XXX**
HR beats/min<br>• highest<br>• lowest	**NUMBER XXX**
RR Breaths/min<br>• highest<br>• lowest	**NUMBER XX**
BP mm of Hg<br>• Systolic<br>Lowest in 24 hours<br>Highest in 24 hours<br>• Diastolic<br>Lowest in 24 hours<br>Highest in 24 hours<br>• Mean<br>Lowest in 24 hours<br>Highest in 24 hours	**NUMBER XXX**
Temp Centigrade<br>• Core<br>• Peripheral	**NUMBER XX.X**
GCS<br>lowest<br>highest	**NUMBER XX**

For peer review only

Blood gas (blood gas parameters in any sample for a 24 hours period)<br>• pH (lowest in 24 hrs)<br>• PaO2 KPa (lowest in 24 hours)<br>• PaCO2 KPa (highest in 24 hours)<br>• Base Excess (from the lowest pH gas result)<br>• Bicarbonate mmol/L (from the lowest pH gas result)	**NUMBERS**<br><br>**pH X.XX**<br>**PaO2 XX.X**<br>**PaCO2 XX.X**<br>**Base Excess XX.X**<br>**Bicarbonate XX.X**
Urine output (UOP)<br>Lowest UOP in any 6 hours over a 24 hours period<br>Diuretic medication Y/N	**NUMBER**<br>**XX.X**
Lactate mmol/L<br>Highest lactate	**NUMBER**<br>**XX.X**
CRP mg/L	**NUMBER XXX.X**
Beta D Glucan pg/ml	**xxx.x**
Hb g/l	**NUMBER XXX**
WCC number / cumm<br>• Lymphocytes<br>• Neutrophils	**NUMBER XXX.X**
Platelets number/cumm	**NUMBER XXXX**
PT seconds	**NUMBER XX**
APTT seconds	**NUMBER XXX**
INR ratio	**NUMBER X.X**
ALT U/L	**NUMBER XXXX**
Bilirubin µmol/L	**NUMBER XXX.X**
Urea mmol/L	**NUMBER XX.X**
Creatinine µmol/L	**NUMBER XXX.X**
Na mmoll/L	**NUMBER XXX**
K mmoll/L	**NUBMER XX.X**
Cl mmoll/L	**NUMBER XXX**
d-Dimers µg/L	**NUMBER XXXXXX**
Ferritin µg/L	**NUMBER XXXXXX**
CXR signs of lower resp infection	**Yes / No**
Other<br>• specify	**entries with free text**

For peer review only

Daily Organ Support 24 hours (00:00 to 24:00) this is done every day, like the information above

Respiratory Yes/No<br>If 'Yes' t*ick relevant box below to select*	• Non Invasive<br>○ High Flow<br>○ CPAP<br>○ BiPAP<br>• Invasive Mechanical ventilation<br>• High Frequency Oscillation
Cardiovascular<br>Vaso active infusions Yes/No, if Yes ...tick relevant box	• Dopamine<br>• Dobutamine<br>• Adrenaline<br>• Noradrenaline<br>• Vasopressin<br>• Milrinone<br>Other – please specify
Haemofiltration/Haemodialysis Yes/No	
ECMO Yes/No	

For peer review only

Subsequent Research blood samples: date and time – DD:MM:YY HH:MM

To derive duration from time of admission: __:__ (hh:mm) and other events such as death and blood cultures

Time point (tp1 to tp7)	Date and time	Clinical Correlation eg: admission; onset of hypotension, cardiac arrest etc.,

Procedures during admission:

Endotracheal Intubation: Y/N

Central Venous Line Insertion: Y/N

Arterial Line Insertion: Y/N

Chest Drain insertion: Y/N

Date of procedure: DD/MM/YYYY

Other:

Please specify

Surgery during PCCU admission: Yes/No DATE TIME DD:MM:YY HH:MM

If yes, please specify:

DATE AND TIME DD:MM:YY HH:MM

To derive relation to admission to PCCU and research sample date and time

Microbiological investigations (includes bacterial, viral, fungal or any other investigations to identify a causative organism for illness)

1. Blood culture: *Provision to input more than one entry for all categories from 1 to 8*

For peer review only

Requested Y/N

If Y, Result: Positive/Negative

If 'Positive' - please specify

If positive is this significant or no significant? Check box.

Free text for the bacterium

DATE AND TIME DD:MM:YY HH:MM

To derive relation to research sample date and time

2. Naso-pharyngeal aspirate for respiratory panel:

Requested: Yes/No

If yes, result:

Influenza A PCR RNA
Influenza B PCR RNA
RSV PCR RNA
Parainfluenza PCR RNA
Rhinovirus/Enterovirus PCR
HMPV PCR RNA
Adenovirus PCR DNA
Mycoplasma PCR DNA
Enterovirus PCR RNA
Coronavirus PCR

Positive/Negative

If 'Positive' – please specify

DATE AND TIME DD:MM:YY HH:MM

To derive relation to research sample date and time

3. COVID-19 test:

Requested: Yes/No

Throat swap: Yes/No

If Yes- Positive/Negative?

non Bronchoscopic Alveolar Lavage (nBAL): Yes/No

If 'Yes' – Positive/Negative?

DATE AND TIME DD:MM:YY HH:MM

To derive relation to research sample date and time

For peer review only

4. Urine MCS

Requested: Yes/No

If yes, result: Positive/Negative

If 'Positive' – please specify

DATE AND TIME DD:MM:YY HH:MM

To derive relation to research sample date and time

5. CSF MCS

Requested: Yes/No

If yes, result: Positive/Negative

If 'Positive' – please specify

DATE AND TIME DD:MM:YY HH:MM

To derive relation to research sample date and time

6. Non Bronchoscopy Alveolar Lavage (NBAL)

Requested: Yes/No

If yes, result: Positive/Negative

If 'Positive' – please specify

DATE AND TIME DD:MM:YY HH:MM

To derive relation to research sample date and time

7. Sputum culture

Requested: Yes/No

If yes, result: Positive/Negative

If 'Positive' – please specify

DATE AND TIME DD:MM:YY HH:MM

To derive relation to research sample date and time

8. Other specimens/Tests

Please specify

For peer review only

(Prompt to add multiple distinct entries)

DATE AND TIME DD:MM:YY HH:MM

To derive relation to research sample date and time

Medications during PCCU admission:

1. Corticosteroid: Yes/No
   If 'Yes', please tick relevant box/boxes to select
   Prednisolone
   Methylprednisolone
   Hydrocortisone
   Dexamethasone

   Other: *please specify*
   Indication

   Dose (Mg/Kg)

   Date started DD/MM/YY
   Date stopped DD/MM/YY
   To derive duration and relation to date of admission to PCCU

2. Antibiotic: Yes/No
   If 'Yes', please tick relevant box/boxes to select

   Co-Amoxiclavulonic acid (Augmentin)
   Cefotaxime
   Ceftriaxone
   Tazocin
   Meropenem
   Azithromycin
   Clarithyromycin

   Other*: please specify*

   Indication:
   Date started: DD:MM:YY
   Date stopped: DD:MM:YY
   To derive duration and relation to date of admission to PCCU

3. Antiviral Yes/No
   If yes, please tick relevant box/boxes to select

For peer review only

Aciclovir
Gancliclovir

Other: *please specify*

Indication:

Date started: DD/MM/YY
Date stopped: DD/MM/YY
To derive duration and relation to date of admission to PCCU

4. Antifungal Y/N
   If 'Yes', please tick relevant box/boxes to select

   Fluconazole
   Ambisome
   Itraconazole

   Other: please specify

   Indication:

   Date started: DD/MM/YY
   Date stopped: DD/MM/YY
   To derive duration and relation to date of admission to PCCU

5. Anti-inflammatory medications : Yes/No
   If 'Yes', please tick relevant box/boxes to select

   Ibuprofen
   Other NSAID
   Other: please specify

   Indication:

   Date started: DD/MM/YY
   Date stopped: DD/MM/YY
   To derive duration and relation to date of admission to PCCU
6. Other medications modifying immunity :: Yes/No
   (Includes chemotherapy or Antibody based medications)
   If 'Yes', please tick relevant box/boxes to select

   Intravenous Immunoglobulin (IVIG)
   Methotrexate
   Vincristine
   Daunorubicin

For peer review only

Infliximab
Anakinra
Tocilizumab

Other: please specify

Indication:

Date Started: DD/MM/YY

Date Stopped: DD/MM/YY

To derive duration and relation to date of admission to PCCU

Clinical Outcome:

1. Duration on mechanical ventilation: __(DD)
2. Duration on Vase-active agents:__(DD)
3. Date of discharge from PCCU: DD/MM/YY
   To derive duration of stay in PCCU: ___ (DD)
4. Alive at discharge: Yes/No
   If No to 4, (clinical outcome is death), Date of death: DD/MM/YY
   To derive duration of stay in PCCU
   To derive duration from research blood samples
   Withdrawal of life support: Yes/No?
5. Transfer to different hospital: Y/N

Confirmed diagnosis at time of discharge from PCCU: Yes/No

(In case of infectious illness – please specify if microbiologically confirmed)

If 'Yes' please specify

(Please include microbiological diagnosis as well as the complications e.g.: exacerbation of asthma secondary to Rhinovirus infection)

This may involve conclusion following Multi-professinal discussions at the time including Infectious Disease specialist, Microbiologist or any other specialists.

Indeterminate diagnosis at discharge from PCCU: Yes/No

:

Final diagnosis available from clinical follow up Yes/No

If 'Yes' please give details

Is the Adjudicator's diagnosis same as the final diagnosis on follow up?

Yes/No

For peer review only

Clinical Phenotyping

Consensus between two clinicians? Yes /No

If no..

FINAL ADJUDICATOR OPINION:

if yes..

CONSENSUS CLINICAL PHENOTYPING:

Infectious Illness: Yes ☐ No ☐

If infectious illness..

please choose:

- Suspected ☐ (or)
- Proven by positive culture, tissue stain, or PCR test ☐ (or)
- a clinical syndrome associated with a high probability of infection ☐

Type of infectious illness:

Not sepsis ☐

Sepsis ☐

If sepsis..

Choose microbiological agent:

- Sepsis with confirmed bacterial infection ☐
- Sepsis with confirmed other microbiological (eg. Viral, fungal) infection ☐
- Sepsis with no microbiological confirmation ☐

Choose severity of sepsis:

- Sepsis ☐
- Sepsis associated organ dysfunction cardiovascular or non-cardiovascular organ dysfunction ☐
- Septic shock ☐

Diagnosis

Non-infectious illness – please state diagnosis ______________________

Sterile inflammatory illness ☐ eg. Trauma (or) Asthma

Non inflammatory illness ☐ eg. DKA or Status Epilepticus

Infectious illness – please state diagnosis _______________________

For peer review only

Adverse events: Yes/No

If 'Yes', please specify details:

Date and Time: DD/MM/YY

(DATE FOR USE ONLY BY SPONSOR)

Please derive duration from admission to PCCU

Was it related to research?

If yes, please specify if expected from research procedures or unexpected

Was the adverse event considered serious? : Yes/No

If yes, please specify details why it was considered serious:

Has the SAE (related) been reported to REC/Sponsor: Yes/No

(Deaths or complications from acute illness are not considered serious adverse events)

For peer review only

Case Report Form (CRF) review tool for clinical phenotyping

Criteria for sepsis

Paediatric consensus conference sepsis criteria, 2005

Presence of Systemic Inflammatory Response Syndrome with suspected or proven infection.

Description of criteria for the PCCS definition of sepsis:

<u>Systemic Inflammatory Response Syndrome (SIRS)</u>

The presence of at least two of the following four criteria, one of which must be abnormal temperature or leukocyte count: This is during any point from first contact with health care professionals or during stay in PCCU.

- Core temperature >38.5C or <36C ☐
- Tachycardia, defined as a mean heart rate of >2SD above normal for age ☐
  Or (for infants, bradycardia < 10th centile for age)
- Mean respiratory rate > 2SD above normal for age or mechanical ventilation for an acute process ☐
- Leucocyte count elevated or depressed for age ☐

Evidence of infection includes positive findings on clinical exam, imaging, laboratory tests (e.g., white blood cells in a normally sterile body fluid, perforated viscus, chest radiograph consistent with pneumonia, petechial or purpuric rash, or purpura fulminans)

Age specific vital signs and laboratory parameters:

Age Group[a]	Heart Rate, Beats/Min[b,c]		Respiratory Rate, Breaths/Min[d]	Leukocyte Count, Leukocytes × $10^3/mm^3$ [b,c]	Systolic Blood Pressure, mm Hg[b,c,e,f]
	Tachycardia	Bradycardia			
0 days to 1 wk	>180	<100	>50	>34	<65
1 wk to 1 mo	>180	<100	>40	>19.5 or <5	<75
1 mo to 1 yr	>180	<90	>34	>17.5 or <5	<100
2–5 yrs	>140	NA	>22	>15.5 or <6	<94
6–12 yrs	>130	NA	>18	>13.5 or <4.5	<105
13 to <18 yrs	>110	NA	>14	>11 or <4.5	<117

Surviving sepsis campaign for sepsis guidelines in children, 2020

- **Defined as per the PCCS 2005 guideline (OR) evidence of *severe bacterial infection leading to life threatening organ dysfunction would be included, even when there is deviation from the specific 2005 criteria above.***

For peer review only - http://bmjopen.bmj.com/site/about/guidelines.xhtml

Study ID:

Consensus between two clinicians Yes /No

If 'No'

Final Adjudicator opinion sought Yes/No

Acute illness: Yes ☐ No ☐

Infectious Illness: Yes ☐ No ☐

If infectious illness, please state:

- Suspected ☐ (or)
- Proven by positive culture, tissue stain, or PCR test ☐ (or)
- a clinical syndrome associated with a high probability of infection ☐

Type of infectious illness:

Not sepsis ☐

Sepsis ☐ If sepsis, please choose one below

- Sepsis with confirmed bacterial infection ☐
- Sepsis with confirmed viral infection ☐
- Sepsis with no microbiological confirmation ☐

Severity of sepsis is defined based on organ function in to

- Sepsis ☐
- Sepsis associated organ dysfunction cardiovascular or non-cardiovascular organ dysfunction ☐
- Septic shock – sepsis with cardiovascular organ dysfunction with evidence of hypotension, need for treatment with vasoactive medication or impaired perfusion. ☐

Diagnosis

Non-infectious illness – please state diagnosis ______________________

Sterile inflammatory illness ☐ eg. Trauma (or) Asthma

Non inflammatory illness ☐ eg. Diabetic Ketoacidosis or prolonged febrile seizure

Infectious illness – please state diagnosis _______________________

Comments:

For peer review only - http://bmjopen.bmj.com/site/about/guidelines.xhtml

3

For peer review only

3

Clinical phenotyping of patients recruited to pSeP/COVID study

Decision flow chart for reviewers v 0.1

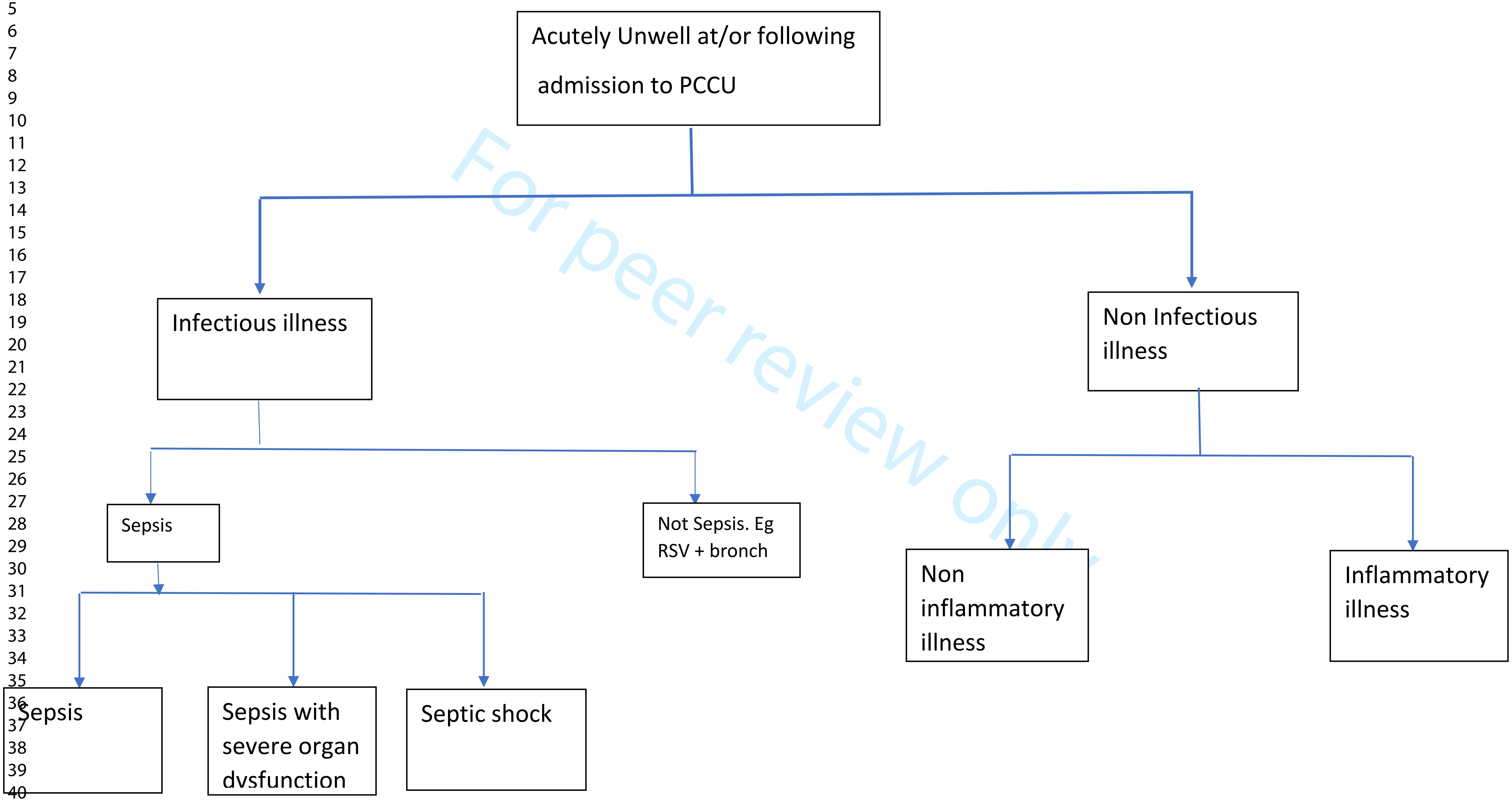

For peer review only

Immune and metabolic markers for identifying and investigating severe Coronavirus disease and Sepsis in children and young people. (pSeP/COVID ChYP study) – Protocol for a prospective cohort study

STROBE Statement—checklist of items that should be included in reports of observational studies

	Item No.	Recommendation	Page No.	Relevant text from manuscript
**Title and abstract**	1	(*a*) Indicate the study's design with a commonly used term in the title or the abstract	1	Included
		(*b*) Provide in the abstract an informative and balanced summary of what was done and what was found		
**Introduction**				
Background/rationale	2	Explain the scientific background and rationale for the investigation being reported		Included under Introduction
Objectives	3	State specific objectives, including any prespecified hypotheses		Included under Methods
**Methods**				
Study design	4	Present key elements of study design early in the paper		Included in Methods
Setting	5	Describe the setting, locations, and relevant dates, including periods of recruitment, exposure, follow-up, and data collection		Included in the Methods
Participants	6	(*a*) *Cohort study*—Give the eligibility criteria, and the sources and methods of selection of participants. Describe methods of follow-up<br>*Case-control study*—Give the eligibility criteria, and the sources and methods of case ascertainment and control selection. Give the rationale for the choice of cases and controls<br>*Cross-sectional study*—Give the eligibility criteria, and the sources and methods of selection of participants		Included in Methods<br>For nested case-control analysis, provided details of the Clinical phenotyping to ascertain cases and controls
		(*b*) *Cohort study*—For matched studies, give matching criteria and number of exposed and unexposed<br>*Case-control study*—For matched studies, give matching criteria and the number of controls per case		N/A
Variables	7	Clearly define all outcomes, exposures, predictors, potential confounders, and effect modifiers. Give diagnostic criteria, if applicable		Provided the details of variables to be measured. Discussed the process for clinical phenotyping

For peer review only - http://bmjopen.bmj.com/site/about/guidelines.xhtml

Immune and metabolic markers for identifying and investigating severe Coronavirus disease and Sepsis in children and young people. (pSeP/COVID ChYP study) – Protocol for a prospective cohort study

Data sources/ measurement	8*	For each variable of interest, give sources of data and details of methods of assessment (measurement). Describe comparability of assessment methods if there is more than one group	Provided details of the sample collection, processing and collection of data. Also provided template of Case Report Form which is used to collect clinical data.
Bias	9	Describe any efforts to address potential sources of bias	Discussed relevant bias and measures to address in the Methods.
Study size	10	Explain how the study size was arrived at	Provided details under section Sample Analysis, Sample size calculation

Continued on next page

For peer review only

Immune and metabolic markers for identifying and investigating severe Coronavirus disease and Sepsis in children and young people. (pSeP/COVID ChYP study) – Protocol for a prospective cohort study

Quantitative variables	11	Explain how quantitative variables were handled in the analyses. If applicable, describe which groupings were chosen and why	Provided the relevant information for the analysis under section Clinical Phenotyping and Sample Analysis
Statistical methods	12	(*a*) Describe all statistical methods, including those used to control for confounding	Provided information in section Methods, Data processing and analysis
		(*b*) Describe any methods used to examine subgroups and interactions	
		(*c*) Explain how missing data were addressed	
		(*d*) *Cohort study*—If applicable, explain how loss to follow-up was addressed<br>*Case-control study*—If applicable, explain how matching of cases and controls was addressed<br>*Cross-sectional study*—If applicable, describe analytical methods taking account of sampling strategy	As the study is aimed to evaluate biomarkers in blood samples, this has not been addressed.
		(*e*) Describe any sensitivity analyses	
**Results**			
Participants	13*	(a) Report numbers of individuals at each stage of study—eg numbers potentially eligible, examined for eligibility, confirmed eligible, included in the study, completing follow-up, and analysed	
		(b) Give reasons for non-participation at each stage	
		(c) Consider use of a flow diagram	
Descriptive data	14*	(a) Give characteristics of study participants (eg demographic, clinical, social) and information on exposures and potential confounders	
		(b) Indicate number of participants with missing data for each variable of interest	
		(c) *Cohort study*—Summarise follow-up time (eg, average and total amount)	
Outcome data	15*	*Cohort study*—Report numbers of outcome events or summary measures over time	
		Case-control study—Report numbers in each exposure category, or summary measures of exposure	
		Cross-sectional study—Report numbers of outcome events or summary measures	

For peer review only

For peer review only - http://bmjopen.bmj.com/site/about/guidelines.xhtml

Immune and metabolic markers for identifying and investigating severe Coronavirus disease and Sepsis in children and young people. (pSeP/COVID ChYP study) – Protocol for a prospective cohort study

Main results	16	(*a*) Give unadjusted estimates and, if applicable, confounder-adjusted estimates and their precision (eg, 95% confidence interval). Make clear which confounders were adjusted for and why they were included
		(*b*) Report category boundaries when continuous variables were categorized
		(*c*) If relevant, consider translating estimates of relative risk into absolute risk for a meaningful time period

Continued on next page

For peer review only

Immune and metabolic markers for identifying and investigating severe Coronavirus disease and Sepsis in children and young people. (pSeP/COVID ChYP study) – Protocol for a prospective cohort study

Other analyses	17	Report other analyses done—eg analyses of subgroups and interactions, and sensitivity analyses
**Discussion**		
Key results	18	Summarise key results with reference to study objectives
Limitations	19	Discuss limitations of the study, taking into account sources of potential bias or imprecision. Discuss both direction and magnitude of any potential bias
Interpretation	20	Give a cautious overall interpretation of results considering objectives, limitations, multiplicity of analyses, results from similar studies, and other relevant evidence
Generalisability	21	Discuss the generalisability (external validity) of the study results
**Other information**		
Funding	22	Give the source of funding and the role of the funders for the present study and, if applicable, for the original study on which the present article is based

*Give information separately for cases and controls in case-control studies and, if applicable, for exposed and unexposed groups in cohort and cross-sectional studies.

Note: An Explanation and Elaboration article discusses each checklist item and gives methodological background and published examples of transparent reporting. The STROBE checklist is best used in conjunction with this article (freely available on the Web sites of PLoS Medicine at http://www.plosmedicine.org/, Annals of Internal Medicine at http://www.annals.org/, and Epidemiology at http://www.epidem.com/). Information on the STROBE Initiative is available at www.strobe-statement.org.

For peer review only - http://bmjopen.bmj.com/site/about/guidelines.xhtml

BMJ Open

Immune and metabolic markers for identifying and investigating severe Coronavirus disease and Sepsis in children and young people. (pSeP/COVID ChYP study) – Protocol for a prospective cohort study

Journal:	*BMJ Open*
Manuscript ID	bmjopen-2022-067002.R1
Article Type:	Protocol
Date Submitted by the Author:	02-Mar-2023
Complete List of Authors:	Oruganti, Sivakumar; University Hospital of Wales, Paediatric Critical Care Unit<br>Rodrigues, Patrícia R.S.; Cardiff University,<br>White, Daniel; Cardiff University<br>Watkins, W John; Cardiff University, Cochrane Institute of Primary Care and Public Health<br>Shapey , Selyf; Noah's Ark Children's Hospital for Wales, Paediatric Critical Care Unit<br>Barrow, Anna; Noah's Ark Children's Hospital for Wales, Paediatric Critical Care Unit<br>al Samsam, Rim; Noah's Ark Children's Hospital for Wales, Paediatric Critical Care Unit<br>Ali, Sara; Noah's Ark Children's Hospital for Wales, Paediatric Critical Care Unit<br>Gajraj, Malcolm; Noah's Ark Children's Hospital for Wales, Paediatric Critical Care Unit<br>Skone, Richard; Noah's Ark Children's Hospital for Wales, Department of Paediatric Intensive Care<br>Jardine, Michelle; Noah's Ark Children's Hospital for Wales, Paediatric Critical Care Unit<br>Evans, Jennifer; Noah's Ark Children's Hospital for Wales, Paediatric Critical Care Unit<br>Struik, Siske; Noah's Ark Children's Hospital for Wales, Paediatric Critical Care Unit<br>Song, Jong; Noah's Ark Children's Hospital for Wales, Paediatric Critical Care Unit<br>Abood, Lloyd; Morriston Hospital<br>Paquete, Barbara; Noah's Ark Children's Hospital for Wales, Paediatric Critical Care Unit<br>Foulkes, Sian; Noah's Ark Children's Hospital for Wales, Paediatric Critical Care Unit<br>Saunders, Benjamin; Noah's Ark Children's Hospital for Wales, Infectious Diseases services for Wales<br>Strang, Angela; Cardiff University<br>Kotecha, Sarah; Cardiff University, Department of Child Health<br>Phillips, Bethan; University Hospital of Wales, Children's and Young Adults Research Unit<br>Evans, Awen; University Hospital of Wales, Children's and Young Adults

	Research Unit<br>Buchanan, Iona; University Hospital of Wales, Children's and Young Adults Research Unit<br>Bowes, Susan; University Hospital of Wales, Children's and Young Adults Research Unit<br>Ali, Begum; University Hospital of Wales, Children's and Young Adults Research Unit<br>Gore, Maya; University Hospital of Wales, Children's and Young Adults Research Unit<br>Thomas-Turner, Rhian; University Hospital of Wales, Children's and Young Adults Research Unit<br>Andrews, Robert; Cardiff University<br>Zaher, Summia; University Hospital of Wales, Department of Obstetrics and Gynaecology<br>Sharma, Simran; Cardiff University, Infection and Immunity; Cardiff and Vale NHS Trust, Women's unit<br>Chakraborty, Mallinath; University Hospital of Wales,<br>Parkinson, Edward; Cardiff University; Cardiff University<br>Liberatore, Federico; Cardiff University<br>Woolley, Thomas; Cardiff University<br>Edkins, Sarah; Cardiff University<br>Davies, Luke C.; Cardiff University<br>Moet, Linda; Cardiff University,<br>McLaren, James E.; Cardiff University<br>Watson, Gareth L.; Cardiff University<br>O'Donnell, Valerie; Cardiff University College of Biomedical and Life Sciences, Centre for Trials Research<br>Hood, Kerry; Cardiff University College of Biomedical and Life Sciences, Centre for Trials Research<br>Ghazal, Peter; Cardiff University, Medical School

**Primary Subject Heading**:	Paediatrics
Secondary Subject Heading:	Infectious diseases, Intensive care
Keywords:	Paediatric intensive & critical care < ANAESTHETICS, INFECTIOUS DISEASES, COVID-19

SCHOLARONE™
Manuscripts

Immune and metabolic markers for identifying and investigating severe Coronavirus disease and Sepsis in children and young people. (pSeP/COVID ChYP study) – Protocol for a prospective cohort study

Sivakumar Oruganti[1,2], Patrícia R.S. Rodrigues[2], Daniel White[2], William John Watkins[2], Selyf Shapey[1], Anna Barrow[1], Rim al Samsam[1], Sara Ali[1], Malcolm Gajraj[1], Richard Skone[1], Michelle Jardine[1], Jennifer Evans[3], Siske Struik[3], Jong Eun Song[1], Lloyd Abood[4], Barbara Paquete[1], Sian Foulkes[1], Benjamin Saunders[3] Angela Strang[2], Sarah J Kotecha[2,10], Bethan Phillips[5], Awen Evans[5], Iona Buchanan[5], Susan Bowes[5], Begum Ali[5], Maya Gore[5] and Rhian Thomas-Turner[5], Robert Andrews[2,] Summia Zaher[2,6], Simran Sharma[2,6], Mallinath Chakraborty[2,7] Edward Parkinson[8], Federico Liberatore[8], Thomas E Woolley[9], Sarah Edkins[2], Luke C. Davies[2], Linda Moet[2], James E. McLaren[2], Gareth L. Watson[10], Valerie O'Donnell[2], Kerry Hood[10], Peter Ghazal[2]

[1]Regional Paediatric Intensive Care Unit, University Hospital of Wales, Cardiff, UK
[2] School of Medicine, Cardiff University, Cardiff, UK
[3]Infectious Disease Service for Wales, Noah's Ark Children's Hospital, Cardiff, UK
[4]Morriston Hospital, Swansea, Wales, UK
[5]Children's and Young Adults Research Unit, University Hospital of Wales, Cardiff ,UK
[6]Department of Obstetrics and Gynaecology, University Hospital of Wales, Cardiff, UK
[7]Regional Neonatal Intensive Care Unit, University Hospital of Wales, Cardiff, UK
[8]School of Computer Science and Informatics, Cardiff University.
[9] School of Mathematics, Cardiff University, Senghennydd Road, Cardiff, UK
[10]Centre for Trials Research, Cardiff University, Cardiff UK

Corresponding Author

Dr Sivakumar Oruganti
Paediatric Critical Care Unit
Noah's Ark Children's Hospital
University Hospital of Wales
Health Park
Cardiff
CF14 4XW
Phone: 02720 746423
Email: siva.oruganti@wales.nhs.uk

For peer review only

ABSTRACT

Introduction

Early recognition and appropriate management of paediatric sepsis are known to improve outcomes. A previous systems biology investigation of the systemic immune response in neonates to sepsis identified immune and metabolic markers that showed high accuracy for detecting bacterial infection. Further gene expression markers have also been reported previously in the paediatric age group for discriminating sepsis from control cases. More recently, specific gene signatures were identified to discriminate between COVID-19 and its associated inflammatory sequelae. Through the current prospective Cohort study, we aim to evaluate immune and metabolic blood markers which discriminate between sepsis (including COVID-19) from other acute illnesses in critically unwell children and young persons, up to 18 years of age.

Methods and analysis

We describe a prospective cohort study for comparing the immune and metabolic whole blood markers in patients with sepsis, COVID-19 and other illnesses. Clinical phenotyping and blood culture test results will provide a reference standard to evaluate the performance of blood markers from the research sample analysis. Serial sampling of whole blood (50μl each) will be collected from children admitted to intensive care and with an acute illness to follow time dependent changes in biomarkers. An integrated lipidomics and RNASeq transcriptomics analyses will be conducted to evaluate immune-metabolic networks that discriminate sepsis and COVID-19 from other acute illnesses. This study received approval for deferred consent.

Ethics and dissemination

The study has received research ethics committee approval from the Yorkshire and Humber Leeds West Research Ethics Committee 2 (reference 20/YH/0214; IRAS reference 250612). Submission of study results for publication will involve making available all anonymised primary and processed data on public repository sites. This study has been registered in the Clinical Trials database register. (NCT04904523*)*

STRENGTHS AND LIMITATIONS OF THIS STUDY

► Multi-modal investigation of molecular biomarkers for the early diagnosis of paediatric sepsis using a minimally invasive low blood volume.

► Identification of host–response molecular patterns associated with various infections (bacterial, viral and fungal).

► Immunophenotyping, immunoassays, lipidomics and functional analysis will be performed during the hospital journey and in cases of suspected sepsis to investigate time-dependent changes in biomarkers of sepsis.

►Potential selection bias towards younger cases

►Single site observational study

For peer review only

INTRODUCTION

Sepsis is a global health problem affecting all age groups. A systematic review of published literature from 1979 to 2016 was carried out to estimate population-based sepsis incidence in neonates and children. Reports from 15 studies in 12 countries showed an aggregate estimate of 48 (95% CI 27–86) sepsis cases and 22 (14–33) severe sepsis cases in children per 100,000 person-years. The population-level estimate for neonatal sepsis was 2202 (95% CI 1099–4360) per 100,000 livebirths, with mortality between 11% and 19%. Extrapolating on a global scale gave an estimated incidence of approximately 3 million cases of sepsis in neonates and 1.2 million cases in children annually. Mortality ranged from 1% to 5% for sepsis and 9% to 20% for severe sepsis.[1]

Definition of sepsis in children

At present, sepsis is defined in adults as life-threatening organ dysfunction resulting from a dysregulated host response to infection [2]. This definition has removed the terms 'Systematic Inflammatory Response Syndrome' (SIRS) and 'severe sepsis' found in previous definitions and highlights organ dysfunction for clinical management. In children, the existing Surviving Sepsis consensus conference definition of sepsis based on the criteria for SIRS in the presence of confirmed or suspected infection is still used for identification of sepsis and clinical research [3]. The recently updated guidance for the management of sepsis in children (2020) recognises the clinical heterogeneity of this illness in children, accounting for life-threatening organ dysfunction [4]. A revised definition of sepsis by the paediatric sepsis definition task force is awaited [5]. Recently we put forward a setpoint mechanism for understanding sepsis. This suggests that *dysregulation* of the host response to infection in sepsis is explainable by regulatory shifts in homeostasis **setpoints.** This is due to negative and positive feedback changes in the concerted behaviour of multiple (immune, metabolic, and neuronal) systems in response to infection. We hypothesise that infants and children have age-dependent metabolic demands and a developing immune setpoint that increases the risk of sepsis [6]. Sepsis can arise from any type of infection - bacterial, viral, fungal, or parasitic [4]. Even though less common in children, COVID-19 can be associated with severe illness. History of infection and age-dependent changes in the immune system give rise to subtle but important differences in response to SARS-CoV-2 infection in children when compared to adults. In the early life and younger years, the innate response is a dominant feature of the immune response to infection. In the case of SARS-CoV-2 infection, children are known to mount a stronger and more vigorous Interferon (IFN) antiviral response than adults, curtailing viral replication at an early stage of infection[7].

Biomarkers in sepsis

Several host inflammatory biomarkers have been suggested to improve the ability for early recognition of sepsis. These include C-Reactive Protein (CRP), Pro-calcitonin (PCT), Interleukin-8, soluble CD14 (presenilin) and triggering receptor expressed on myeloid cells (sTREM-1) that have all been evaluated without conclusive results [8]. It is important to recognise that sepsis is a complex pathophysiological process comprising an immune response which interacts with the body's metabolic and physiologic functions. A profile of multiple markers capturing the complexity of the pathophysiological response would be better than an individual marker to detect and study the disease process [9]. In neonatal sepsis, it has been shown that the inclusion of metabolic markers as well as adaptive immune responses with innate immune signal changes, increased the accuracy for early recognition of sepsis [10].

For peer review only

These complex molecular changes during sepsis correlate with altered gene expression[11] and in some infectious illnesses, there was a unique signature alteration in gene expression with a demonstrable value in understanding pathogenicity [12-14]. Gene expression markers have been evaluated to identify bacterial infections in febrile children [15] and differences in transcriptomic profiles in blood have been used to discriminate clinical phenotypes and sub-groups of sepsis [16,17].

Coronavirus disease (COVID-19) in children and young people admitted to paediatric intensive care

Infection with Severe Acute Respiratory Syndrome-Coronavirus 2 (SARS-CoV-2) is mild or asymptomatic in the majority of children [18,19]. The proportion of children presenting to the hospital who have severe COVID-19 and require admission to intensive care has been variable ranging from 1.75% in China [20] to a higher rate of 9.7% to 28% in the western world [21-23]. The clinical risk factors for severe COVID-19 in children have been reported to be age over 10 years, black and Asian ethnic groups, co-morbidities and obesity [23,24]. The difference in the severity of COVID-19 in children, when compared to adults and the elderly, has been studied extensively. Various factors to explain this difference have been reported and include vasculopathy, the density of Angiotensin-Converting Enzyme 2 (ACE 2) receptors, age-based differences in coagulation profiles and immune responses [25], and heightened antiviral IFN response (Yoshida 2021) [7]. Paediatric Inflammatory Multisystem Syndrome Temporally related to SARS-CoV-2 (PIMS-TS) also called Multisystem Inflammatory Syndrome in Children (MIS-C) is a hyperinflammatory syndrome seen in some children following contact with SARS-CoV-2. Two phenotypes have been described – high fevers with severe gastrointestinal symptoms; and shock with myocardial dysfunction and Kawasaki disease-like clinical features. Research to evaluate the pathophysiology of PIMS-TS reported an abnormal immune response mediated by Super Antigen (SAg) activity of the SARS-CoV-2 S glycoprotein [26]. It has also been associated with immune markers related to endothelial dysfunction in children[27]. Through this study, we hope to compare immune responses in children with COVID-19, PIMS-TS and sepsis.

Study methods for molecular and cellular analysis

The studies so far have been restricted to using transcriptomic analysis for an observational case-control methodology to identify differentially expressed genes in children with sepsis (cases) when compared to children without an acute illness (controls). Evaluation of differentially expressed genes in a particular illness, when compared to healthy controls, helps in understanding the biological pathways and pathophysiology of the disease processes. However, the use of healthy controls in studies aiming to identify gene expression markers that discriminate a particular disease from other illnesses may lead to spectrum bias and fail to account for time-resolved differences [28]. Further, the use of other modal data inclusive of metabolites, in particular lipids, proteomic, cellular and clinical observations allow for a more complete picture of the underlying pathophysiology and diagnostic algorithm development. We propose a prospective cohort methodology to investigate the differentially expressed genes over time in children with sepsis and other acute illnesses.

METHODS

Hypothesis and specific aims

A neonatal-sepsis classifier integrating immune and metabolic pathways was able to discriminate neonates with bacterial infections and sepsis with high accuracy [10]. We

For peer review only - http://bmjopen.bmj.com/site/about/guidelines.xhtml

hypothesise that the integrative tripartite immune-metabolic pathways represented by differentially expressed genes between sepsis and other illnesses would discriminate bacterial infection causes of sepsis from COVID-19 illness and other acute non-infectious illnesses. The immune-metabolic pathways vary between different age groups in children under 18. Our specific aims include:

- Compare immune and metabolic markers which discriminate sepsis and COVID-19 from other acute non-infectious illnesses in children admitted to PICU.
- Investigate the evolution of immune responses in children with sepsis and COVID using serial samples.
- Evaluate the utility of gene expression markers to discriminate heterogeneous sub-groups of sepsis and other critical illnesses with the help of deep clinical phenotyping.
- Understand physiological processes associated with COVID-19 and PIMS-TS and how they differ from bacterial sepsis aetiology.
- Explore age-dependent differences in systemic host-response and determine whether these differences are associated with the risk of disease severity to infection.

We plan to explore the feasibility of objectively defining the balance between immune tolerance and resistance in general, as well as in different paediatric age groups. This is through investigating pathway biology response to sepsis and multi-omic analyses.

This is a prospective cohort study with a nested case-control analysis. The start date was 30/06/2020 and the proposed end date is 01/06/2023.

Inclusion criteria
Children or young people under 18 years who

1. Are admitted to paediatric critical care unit and
2. Have an acute illness including trauma and
3. Have routine blood tests as part of their clinical care

(or)
Children or young people under 18 years who are admitted to paediatric wards with confirmed COVID-19 illness or PIMS-TS.

Exclusion criteria
1. Admitted to hospital for social reasons without an acute illness.

2. Declined consent by Parents or carers with legal responsibility, or by competent young person

3. Admitted to PCCU electively without an acute illness

4. Direct clinical care team not able to provide research information in a language appropriate for non-English/Welsh speaking participants

Patient/Family journey (please see Figure 1, Patient Recruitment and Blood Sample analysis workflows)
Any child or young person with an acute infectious illness or a non-infectious illness such as trauma on admission to PCCU will be eligible for participation in the Study. All children will

For peer review only

be screened by the direct clinical care team and research nurse. Research blood samples will be collected in those who are eligible Data collected will include clinical and laboratory information essential for clinical characterisation including clinical profile, the severity of illness and clinical outcomes. There is no follow-up planned nor repeat blood samples following discharge from the hospital. Anonymous clinical data during the index hospital admission will be collected.

Research blood sample collection

Research blood sample will be collected at admission or onset of an acute illness in eligible children on paediatric intensive care unit. In COVID or PIMS-TS patients on paediatric wards and who do not need intensive care, research blood samples will be collected once consent is obtained. Further blood samples may be collected but only alongside clinical blood samples and at least 24 hours apart during the illness, aiming to collect a final sample during recovery from organ failure. All research blood samples will only be collected along with blood samples required for routine clinical care.

Case definitions and group stratification criteria

We aim to retrospectively carry out deep clinical phenotyping of all patients recruited for the Study using anonymous data from the Case Report Forms(CRF) (Appendix 1). Two clinicians will independently review the data and allocate a diagnosis to all recruited patients broadly into 'Infectious illness', and 'Non-infectious illness' with further specific categorization into various specific illnesses including COVID-19 and Sepsis (Appendix 2 & 3 - Clinical Phenotyping tool and Clinical phenotyping flow chart respectively). In case of any disagreement among the two clinicians, a third clinician will provide a final decision. This forms the reference standard for the classification of illness to aid the interpretation of the research sample analysis results.

The current practice is to isolate all children admitted to the paediatric critical care unit and wards until their SARS-CoV-2 PCR test result is known. Only those cases that meet the clinical phenotype of COVID-19 illness will be categorised as COVID-19[29]. Patients who are coincidentally positive for SARS-CoV-2 but identified with other illnesses on clinical phenotyping, will not be categorised as COVID-19.

As the revisions to the definition of sepsis are pending, clinical phenotyping will be broadly based on the surviving sepsis consensus definition (2005). Patients will be included if they meet 2 or more criteria of Systemic Inflammatory Response Syndrome (SIRS), with at least one criteria related to white cell count or temperature, and with confirmed or strongly suspected to have an infection [3]. There have been algorithms used previously to identify bacterial infections [30,31]. In this Study we plan to evaluate sepsis due to any aetiology including fungal or viral infections. The clinical phenotyping will aid in categorising patients into descriptive clinical clusters which we believe would have more utility in studying differences in immune metabolic biological pathways between sepsis due to viral, bacterial or fungal sepsis or where there is no microbiological agent identified. The process aims to report those cases as sepsis, which may deviate from the 2005 consensus definition, but have strongly suspected or confirmed infection and life threatening organ dysfunction as described in the latest Surviving Sepsis consensus guidance statement [4]. In addition, we can study the differences in sepsis as a whole in comparison with other acute illnesses.In those patients with confirmed infections and who do not meet the SIRS criteria for sepsis deep clinical phenotyping will help us in evaluating the immune and metabolic markers based on patient outcomes such as severity of illness and organ dysfunction.

Recruitment and consent

Recruitment of eligible children admitted to the paediatric critical care unit meets the criteria for emergency research as we need to collect research blood samples soon after admission to the critical care unit along with routine blood tests and it would be inappropriate to approach parents or legal guardians for consent [32]. We have ethics approval for research before consent and collection of multiple samples if the research team find parents not ready to make an informed choice to consent for research. Consultee advice will be sought in patients who are 16 and 17 years old who lack capacity or are not able to make an informed choice due to the critical illness.

In patients admitted to wards with confirmed COVID-19 or PIMS-TS, consent will be obtained after appropriate information sharing before research sample collection at the time of routine clinical blood sampling.

Patient and Public involvement

The study group have engaged with representative members of public for the design of the study. Specific areas where this has led to incorporation of ideas and feedback are listed below: :

1. Justification for the Study: Early identification of severe infectious illness will provide a diagnosis and information on prognosis will help in counselling and communicating with patients, families and parents.
2. Representatives of parents have approved of the methods involving research before consent as this is considered research in an emergency setting. The feedback stressed the importance of timing in the approach to parents of prospective research participants, to allow information sharing and consenting in such a way it causes minimum distress. We agreed that the timing for the approach should be based on the parent's and patients' ability to understand the research and not based on the duration from the collection of the first sample.
3. In view of the importance of the appropriate timing for informed consent, it was also agreed that we could take further samples before consent. This was only in a situation where the direct clinical care team and research nurses felt that the parents may be emotionally not ready for the approach or the prospective research participant was severely unwell.
4. Representative members of the public were involved in drafting the information sheets for parents, carers and older children. The final drafts were approved as appropriate for sharing information with parents and patients at the time of initial approach and consenting. Even though feedback about the information provided was incorporated, the information could not be shortened due to regulatory requirements.
5. Feedback from patient groups through Wales Gene Park included the research methods and information shared with families and legal guardians. Also included specific information related to Next Generation Sequencing in the Study. The feedback was supportive of the methods used and suggestions were incorporated into the Information sheets.

Peer review and feedback were sought through the UK Paediatric Intensive Care Society – Study Group as well as during a previous grant application.

SAMPLE PROCESSING AND DATA ANALYSIS

For peer review only

Sample storage and analysis (please see Figure 1)

The blood samples, at clinical sites, will be stored at –20°C until transferred to the laboratory for processing, where they are stored at -80°C, once consent is obtained.

Blood samples transferred to the laboratory will be processed in batches and initially retained by the pSeP team onsite at Cardiff University in a dedicated lock-secured freezer. For long-term storage and access for future research, samples will be housed by the Cardiff Biobank. For the transcriptomic and lipidomic analysis, the blood is mixed with the stabilising reagent potassium amyl xanthate (PAX) in the collection tubes, all cells are immediately lysed and are not considered to be human tissue. The second tube will be stored as an EDTA-whole blood sample (with or without a stabiliser for cellular phenotyping) and after cell, and targeted proteomics and metabolomics analyses will be banked for future use, for validating future diagnostic platforms.

We aim to apply a systems biology multi-omic analysis of blood samples. This will involve using microarray and RNAseq methodology for probing the transcriptome of whole blood. While for specific immune cell characterisation, we will use techniques such as the Cellular Indexing of Transcriptomes and Epitopes by Sequencing (CITE-Seq) methods that perform RNAseq along with quantitative and qualitative information on surface proteins at a single-cell level. For metabolite and proteomic analyses, we will use the methods of liquid chromatography with tandem mass spectrometry (LC-MS/MS) in a targeted high-throughput manner, and we will use quantitative lipidomic profiling (LC-MS/MS) for comprehensive screening of specific pathways such as the complement system.

Clinical data

Case Report Forms will be used for collecting anonymous clinical data and also containing the clinical classifier (Figure1) from clinical phenotyping (Appendix 1)

Sample size calculation for transcriptomic analysis using RNAseq

We aim to recruit 250 patients with acute illnesses admitted to our paediatric intensive care unit. Extrapolating the results of previous reports on the prevalence of severe sepsis in the paediatric intensive care population, we estimate that there will be around 25 patients with sepsis included in our study [33,34]. For calculating the power and Type 1 error (α), we refer to the Negative Binomial model for biological variability estimations in human samples used by Hart et al (2013) [35]. Using an RNAseq minimum read depth of 30 million reads per sample and a liberal coefficient of variability (σ) of 0.6, we calculate that to achieve a Power(β) of 0.8 and Type 1 error (α) of 0.01, we would need around 20 samples in each group for a two-fold change in differentially expressed genes.

Data processing and analysis

Paired-end reads from RNAseq Illumina sequencing will be trimmed and processed for quality control [36,37], before mapping to the human genome using Spliced Transcript Alignment to a Reference (STAR) [38]. Once we have read counts per gene/trancript, we plan to use R Language and Environment for Statistical Computing [39] followed by quality control and data processing using Bioconductor packages [40]. A per-gene hypothesis of differential average expression will be tested using a negative binomial generalised linear model—DESeq2 package and resulting p-values will be controlled for multiple testing using the Benjamini-Hochberg method [41]. For classification, a variety of machine learning and statistical pathway biology approaches, as described in Smith et al. 2014 [42], will be used. Pathway analyses will be carried out step-wise using a pathway biology approach, becoming more focused. For metabolomic and proteomic data, absolute concentrations (determined by liquid chromatography Mass Spectrometry LC-MS/MS) of analytes in extracts from blood samples

For peer review only

will apply validated tools such as MetaboAnalyst 4.0 [43]. Further multivariate statistical testing (PCA, PLSDA, Random Forest, ANOVA) using group assignment derived from clinical phenotyping (types of sepsis and between sepsis and non-septic controls), will also be used to determine metabolites or proteins that are significantly different in abundance between clinical phenotypes. Single cell analyses (CITE-seq) using surface protein and RNA libraries (10x Genomics) and next-generation sequencing will be multiplexed by cell "hashing" [44]. Demultiplexed sequencing data will be aligned to the reference transcriptome using CellRanger (10x Genomics) and the number of unique molecular identifiers (UMI) per cell will be quantified. Computational analyses and quality control will be performed using R packages including Seurat to integrate hashtag, protein and RNA libraries whilst also enabling demultiplexing of donors, doublet detection and cell clustering [45].
For the final pathway-biomarker assessment of the predictive success of the model, Receiver Operator Characteristics (ROC) will be applied.

DISCUSSION

Through this a prospective observational cohort study, we plan to evaluate immune metabolic biological pathways in children with acute illnesses admitted for paediatric critical care. The aim is to evaluate the differences in immune and metabolic pathways in children with sepsis and other illnesses including COVID-19 in children and young people. The use of the cohort design will help in recruiting children of different age groups from birth to under 18 years admitted to paediatric critical care unit with acute illnesses. The subsequent nested case-control analysis of different clinical groups from the cohort matched for age will help to identify differentially expressed genes among the different clinical phenotypes such as COVID-19 and confirmed sepsis with microbiological confirmation, COVID-19 and confirmed other viral infections, PIMS-TS and confirmed viral infection, PIMS-TS and sepsis with confirmed microbiological confirmation, sepsis with microbiological confirmation and confirmed viral infection. The initial analysis in a discovery cohort will be followed by cross-validation in a subsequent validation cohort.Feedback from parents and patient representatives has highlighted the importance of paediatric research for early identification of infections with subsequent poor outcomes or need for organ support. We hope that this study will help in planning future studies addressing this need. We plan to include children with polytrauma as this is considered an example of sterile inflammation.

DISCLOSURE OF INTEREST

All the authors declare there is no conflict of interest.

FUNDING

The study has received funding from the Welsh Government-EU ERDF for Ser Cymru II programme grant (East Wales ERDF Programme grant number 80762-CU-106) for Project Sepsis (PG). PR was supported in part by MRC (R520481) to PG.

For peer review only - http://bmjopen.bmj.com/site/about/guidelines.xhtml

ETHICS APPROVAL AND DISSEMINATION

Ethical approval was obtained from the Wales Research Ethics Committee 2 (IRAS project ID250612, REC ref: 20/YH/0214). Operational approval was received from Health and Care Research Wales and local resource and capacity were supported by the Research and Development Department of the Cardiff and Vale University Health Board. The study is sponsored by Cardiff University. The sponsor or funder does not have any role in the study design data collection or analysis. Submission of study results for publication will involve making available all anonymised primary and processed data on public repository sites. All study results will be presented at national and international conferences and published in peer-reviewed open-access journals.

Author contributors

SO (Principal Investigator): conceptualized the study and contributed to design, engagement with representative members of public, sample size calculation, recruitment of patients, data acquisition, analysis, interpretation of results, writing and editing the manuscript;
PRSR: Investigation, Methodology, Visualization;
DW: Investigation, Methodology;
WJW contributed to sample size considerations and data analysis;
SS[1], AB, RA, SA, MG, RS, MJ, JE, SS[2], JES, LA, BP, SF and BS are involved in the recruitment of patients, data acquisition, clinical phenotyping, analysis and writing of the manuscript;
AS: Ethics, Resources, Data Curation, Project administration (Project Manager);
SJK: Governance and Study administration;
BP, AE, IB, SB, BA, MG and RT-T contributed to drafting data collection forms, sample storage methods, patient recruitment, data collection and governance.
RA: Formal analysis;
SZ and SS: Contributed to patient information and consenting;
MC: Contributed to considerations around Methodology, Consenting, Sample collection procedures and editing the manuscript;
EP and FL: Quality Assurance, Quality Control and Machine Learning analyses;
TEW: Mathematical modelling and testing setpoint hypothesis;
LCD: Investigation, Methodology;
SE: Resources, Investigation, Project administration, Methodology;
LM: Investigation, Methodology;
JEM: Investigation, Methodology;
GLW and KH: Electronic Case Report Form and Data Management;
VO: Methodology, Resources.
PG (Chief Investigator) contributed to conceptualization, design, engagement with representative members of public, sample analysis methods, data analysis, interpretation of results, writing and editing of the manuscript.

Non author contributors

Helen Thomas, Benjamin Beach and Sarra Beach (representing parents and members of public) contributed to consent methods and information for patients, families and parents.

For peer review only

Representative members of public in Project Sepsis and Wales Gene Park contributed feedback on consent methods and information for patients, families and parents;
Prof Mark Peters (Consultant Paediatric Intensivist, Great Ormond Street Hospital) : Through the UK Paediatric Intensive Care Society Study Group, Prof Peters provided feedback on Study design.

REFERENCES

1. Fleischmann-Struzek C, Goldfarb DM, Schlattmann P, Schlapbach LJ, et al. The global burden of paediatric and neonatal sepsis: a systematic review. Lancet Respir Med. 2018 Mar;6(3):223-230. doi: 10.1016/S2213-2600(18)30063-8.

2. Singer M, Deutschman CS, Seymour CW, et al. The Third International Consensus Definitions for Sepsis and Septic Shock (Sepsis-3). JAMA. 2016;315(8):801-810. doi:10.1001/jama.2016.0287

3. Goldstein B, Giroir B, Randolph, A; International pediatric sepsis consensus conference: definitions for sepsis and organ dysfunction in pediatrics. Pediatr Crit Care Med 2005;6(1):2-8. doi: 10.1097/01.PCC.0000149131.72248.E6.

4. Weiss SL, Peters MJ, Alhazzani W, et al. Surviving Sepsis Campaign International Guidelines for the Management of Septic Shock and Sepsis-Associated Organ Dysfunction in Children. Pediatr Crit Care Med. 2020 Feb;21(2):e52-e106. doi: 10.1097/PCC.0000000000002198.

5. Menon K, Schlapbach LJ, Akech S, et al. Pediatric Sepsis Definition-A Systematic Review Protocol by the Pediatric Sepsis Definition Taskforce. Crit Care Explor. 2020 Jun 11;2(6):e0123. doi: 10.1097/CCE.0000000000000123.

6. Peter Ghazal, Patricia R.S. Rodrigues, Mallinath Chakraborty, Siva Oruganti, Thomas E. Woolley, Challenging molecular dogmas in human sepsis using mathematical reasoning, eBioMedicine, Volume 80, 2022. https://doi.org/10.1016/j.ebiom.2022.104031.

7. Yoshida, M., Worlock, K.B., Huang, N. et al. Local and systemic responses to SARS-CoV-2 infection in children and adults. Nature (2021). https://doi.org/10.1038/s41586-021-04345-x

8. Marshall JC, Vincent JL, Fink MP, Cook DJ, Rubenfield G, Foster D et al. Measures, markers, and mediators: Towards a staging system for clinical sepsis. Crit Care Med 2003; 31:1560 –1567. DOI:10.1097/01. CCM. 00000065186.67848.3A

9. Marshall JC, Reinhardt K for the International Sepsis Forum. Biomarkers in Sepsis. Crit Care Med 2005;3(7):2290-98. DOI: 10.1097/CCM.0b013e3181a02afc.

10. . Smith CL, Dickinson P, Forster T, Craigon M, Ross A, Khondoker MR et al. Identification of a human neonatal immune-metabolic network associated with bacterial infection. Nature Communications. 2014;5:4649. DOI: 10.1038/NCOMMS5649.

11. Prucha M, Ruryk A, Boriss H, Moller E, Zazula R, Herold I et al. Expression profiling: toward an application in sepsis Studies. Shock 2004;22(1);29–33. DOI: PMID: 15201698.

12. Ramilo O, Allman W, Chung W, Mejias A, Ardura M, Glaser C. et al. Gene expression patterns in blood leukocytes discriminate patients with acute infections. Blood 2007; 109(5): 2066–2077. DOI:10.1182/blood-2006-02-002477.

13. Fjaerli H O, Bukholm G, Krog A, Skjaeret C, Holden M, Nakstad B. Whole blood gene expression in infants with respiratory syncytial virus bronchiolitis. BMC Infect. Dis 2006;6:175. DOI: 10.1186/1471-2334-6-175.

14. Ardura M I, Banchereau R, Mejias A, Di Pucchio T, Glaser C, Allantaz et al. Enhanced monocyte response and decreased central memory T cells in children with invasive Staphylococcus aureus infections. PLoS ONE 2009; 4(5), e5446. DOI: 10.1371journal.pone..0005446.

15. Herberg JA, Kaforou M, Wright VJ, et al; IRIS Consortium. Diagnostic Test Accuracy of a 2-Transcript Host RNA Signature for Discriminating Bacterial vs Viral Infection in Febrile Children. JAMA. 2016 Aug 23-30;316(8):835-45. doi: 10.1001/jama.2016.11236.

16. Sweeney TE, Azad TD, Donato M, et al. Unsupervised Analysis of Transcriptomics in Bacterial Sepsis Across Multiple Datasets Reveals Three Robust Clusters. Crit Care Med. 2018 Jun;46(6):915-925. doi: 10.1097/CCM.0000000000003084.

17. Wong HR, Cvijanovich NZ, Allen GL, et al. Validation of a gene expression-based subclassification strategy for pediatric septic shock. Crit Care Med. 2011 Nov;39(11):2511-7. doi: 10.1097/CCM.0b013e3182257675.

18. Li B, Zhang S, Zhang R, Chen X, Wang Y, Zhu C. Epidemiological and Clinical Characteristics of COVID-19 in Children: A Systematic Review and Meta-Analysis. Front Pediatr. 2020 Nov 2;8:591132. doi: 10.3389/fped.2020.591132.

19. Otto WR, Geoghegan S, Posch LC, et al. The Epidemiology of Severe Acute Respiratory Syndrome Coronavirus 2 in a Pediatric Healthcare Network in the United States. J Pediatric Infect Dis Soc. 2020 Nov 10;9(5):523-529. doi: 10.1093/jpids/piaa074.

20. Lu X, Zhang L, Du H, et al; Chinese Pediatric Novel Coronavirus Study Team. SARS-CoV-2 Infection in Children. N Engl J Med. 2020 Apr 23;382(17):1663-1665. doi: 10.1056/NEJMc2005073.

21. Tagarro A, Epalza C, Santos M, Sanz-Santaeufemia FJ, Otheo E, Moraleda C, Calvo C. Screening and Severity of Coronavirus Disease 2019 (COVID-19) in Children in Madrid, Spain. JAMA Pediatr. 2020 Apr 8:e201346. doi: 10.1001/jamapediatrics.2020.1346.

22. Verma S, Lumba R, Dapul HM, et al. Characteristics of Hospitalized Children With SARS-CoV-2 in the New York City Metropolitan Area. Hosp Pediatr. 2021 Jan;11(1):71-78. doi: 10.1542/hpeds.2020-001917.

For peer review only

23. Ouldali N, Yang DD, Madhi F, et al,; investigator group of the PANDOR study. Factors Associated With Severe SARS-CoV-2 Infection. Pediatrics. 2021 Mar;147(3):e2020023432. doi: 10.1542/peds.2020-023432.

24. Shekerdemian LS, Mahmood NR, Wolfe KK, et al; International COVID-19 PICU Collaborative. Characteristics and Outcomes of Children With Coronavirus Disease 2019 (COVID-19) Infection Admitted to US and Canadian Pediatric Intensive Care Units. JAMA Pediatr. 2020 Sep 1;174(9):868-873. doi: 10.1001/jamapediatrics.2020.1948.

25. Zimmermann P, Curtis N. Why is COVID-19 less severe in children? A review of the proposed mechanisms underlying the age-related difference in severity of SARS-CoV-2 infections Arch Dis Child 2021;106:429–439. . doi:10.1136/archdischild-2020-320338

26. Rivas MN, Porritt RA, Cheng MH, et al. COVID-19 Associated Multisystem Inflammatory Syndrome in Children (MIS-C): a novel disease that mimics Toxic Shock Syndrome. The superantigen hypothesis, Journal of Allergy and Clinical Immunology (2020), doi: 10.1016/j.jaci.2020.10.008.

27. Borgel D, Chacron R, Grimaud M, et al. Endothelial Dysfunction as a Component of Severe Acute Respiratory Syndrome Coronavirus 2–Related Multisystem Inflammatory Syndrome in Children With Shock. Crit Care Med. 2021 Nov 1;49(11):e1151-e1156. doi: 10.1097/CCM.0000000000005093.

28. Lijmer JG, Mol BW, Heisterkamp S, et al. Empirical evidence of design-related bias in studies of diagnostic tests. JAMA. 1999 Sep 15;282(11):1061-6. doi: 10.1001/jama.282.11.1061. Erratum in: JAMA 2000 Apr 19;283(15):1963. PMID: 10493205.

29. Docherty AB, Harrison EM, Green CA, et al; ISARIC4C investigators. Features of 20 133 UK patients in hospital with covid-19 using the ISARIC WHO Clinical Characterisation Protocol: prospective observational cohort study. BMJ. 2020 May 22;369:m1985. doi: 10.1136/bmj.m1985.

30. Downes KJ, Weiss SL, Gerber JS, et al; Centers for Disease Control and Prevention Epicenters Program. A Pragmatic Biomarker-Driven Algorithm to Guide Antibiotic Use in the Pediatric Intensive Care Unit: The Optimizing Antibiotic Strategies in Sepsis (OASIS) Study. J Pediatric Infect Dis Soc. 2017 Jun 1;6(2):134-141. doi: 10.1093/jpids/piw023.

31. Herberg JA, Kaforou M, Wright VJ, et al; IRIS Consortium. Diagnostic Test Accuracy of a 2-Transcript Host RNA Signature for Discriminating Bacterial vs Viral Infection in Febrile Children. JAMA. 2016 Aug 23-30;316(8):835-45. doi: 10.1001/jama.2016.11236. Erratum in: JAMA. 2017 Feb 7;317(5):538.

32. Health Research Authority. Planning and Improving Research-Policies, standards and legislation: Research in emergency settings. https://www.hra.nhs.uk/planning-and-improving-research/policies-standards-legislation/research-emergency-settings/ (Date accessed: 05/12/2021).

33. Ruth A, McCracken CE, Fortenberry JD, et al. Pediatric severe sepsis: current trends and outcomes from the Pediatric Health Information Systems database. Pediatr Crit Care Med. 2014 Nov;15(9):828-38. doi: 10.1097/PCC.0000000000000254. PMID: 25226500.

34. Weiss SL, Fitzgerald JC, Pappachan J, et al; Sepsis Prevalence, Outcomes, and Therapies (SPROUT) Study Investigators and Pediatric Acute Lung Injury and Sepsis Investigators (PALISI) Network. Global epidemiology of pediatric severe sepsis: the sepsis prevalence, outcomes, and therapies study. Am J Respir Crit Care Med. 2015 May 15;191(10):1147-57. doi: 10.1164/rccm.201412-2323OC. Erratum in: Am J Respir Crit Care Med. 2016 Jan 15;193(2):223-4. PMID: 25734408

35. Hart SN, Therneau TM, Zhang Y, Poland GA, Kocher JP. Calculating sample size estimates for RNA sequencing data. J Comput Biol. 2013 Dec;20(12):970-8. doi: 10.1089/cmb.2012.0283. Epub 2013 Aug 20. PMID: 23961961.

36. Trim Galore: A wrapping tool around Cutadapt and FastQC to consistently apply quality and adapter trimming to FastQ files, with some extra functionality for Mspl-digested RRBS-type libraries. (https://www.bioinformatics.babraham.ac.uk/projects/trim_galore/)

37.FastQC: A quality control tool for high throughput sequence data. Available at https://www.bioinformatics.babraham.ac.uk/projects/fastqc/

38. STAR: ultrafast universal RNA-seq aligner, Alexander Dobin Carrie A. Davis Felix Schlesinger Jorg DrenkowChris Zaleski Sonali Jha Philippe Batut Mark ChaissonThomas R. Gingeras, Bioinformatics, Volume 29, Issue 1, 1 January 2013, Pages 15–21,

39. R Core Team. *R: A Language and Environment for Statistical Computing*. R Foundation for Statistical Computing (2016). Available from http:// www.R-project.org.

40. Huber W, Carey VJ, Gentleman R, et al. Orchestrating high-throughput genomic analysis with Bioconductor. Nat Methods 2015;12(2):115-21. doi: 10.1038/nmeth.3252

41. Benjamini Y, Hochberg Y Controlling the false discovery rate: a practical and powerful approach to multiple hypothesis testing. J R Stat Soc B. 1995;57:289–300

42. Smith CL, Dickinson P, Forster T, et al. Identification of a human neonatal immune-metabolic network associated with bacterial infection. Nat Commun 2014;5:4649. doi: 10.1038/ncomms5649

43. Chong J, Wishart DS, Xia J. Using MetaboAnalyst 4.0 for Comprehensive and Integrative Metabolomics Data Analysis. Current Protocols in Bioinformatics 2019;68(1):e86. doi: 10.1002/cpbi.86.

44. Stoeckius M, Zheng S, Houck-Loomis B, et al. Cell Hashing with barcoded antibodies enables multiplexing and doublet detection for single cell genomics. *Genome Biology* 2018;19(1):224. doi: 10.1186/s13059-018-1603-1

45. Butler A, Hoffman P, Smibert P, et al. Integrating single-cell transcriptomic data across different conditions, technologies, and species. *Nature Biotechnology* 2018;36(5):411-20. doi: 10.1038/nbt.4096

Figure legend

Figure 1: Patient recruitment and Blood Sample analysis workflows

For peer review only

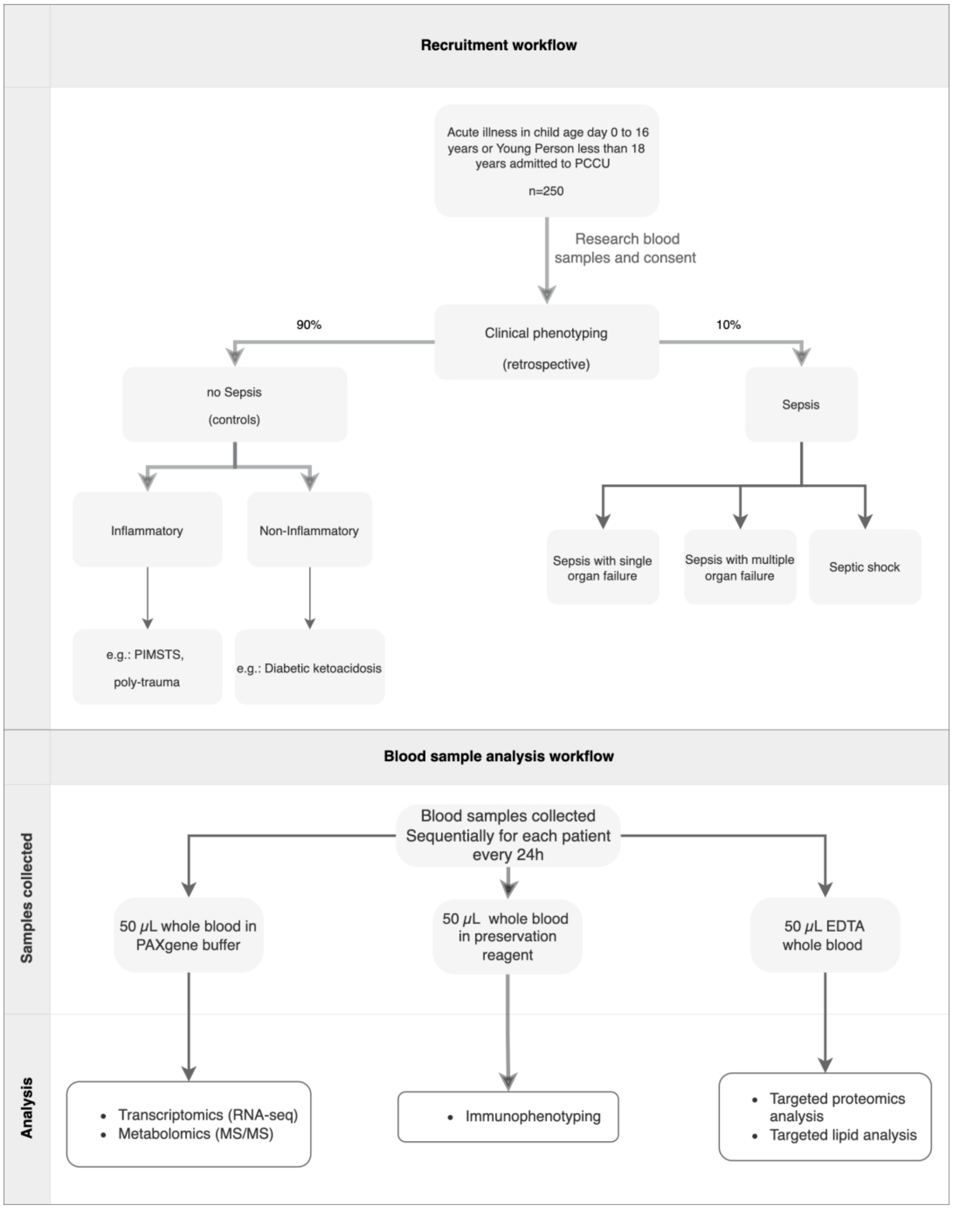

Recruitment and sample analysis workflow

802x1017mm (72 x 72 DPI)

For each patient, free text field 300-400 words.

Once data are input, can be reedited in line with new diagnosis.

<table>
<tr><td>Research ID number</td><td colspan="5"></td></tr>
<tr><td>Age (in years and months)</td><td colspan="5">To encode formula to calculate age upon input of date of birth (for confidentiality)</td></tr>
<tr><td>Age</td><td><1</td><td>2-5</td><td>6-12</td><td>13-15</td><td>16-17</td></tr>
<tr><td>Sex</td><td>M</td><td colspan="2">F</td><td colspan="2">Indeterminate</td></tr>
<tr><td rowspan="3">Ethnicity</td><td>White</td><td colspan="2">Black</td><td colspan="2">West Asian</td></tr>
<tr><td>East Asian</td><td colspan="2">South Asian</td><td colspan="2"></td></tr>
<tr><td colspan="5">Other – please specify</td></tr>
<tr><td>Weight (kgs)</td><td colspan="5"></td></tr>
<tr><td>Weight centile</td><td colspan="5">< 0.4
0.4 to 2
2 to 9
9 to 75
75 to 91
91 to 99.6
>99.6</td></tr>
</table>

Inclusion Criteria

Admitted to PCCU<br>(Or)<br>Admitted to ward with confirmed COVID positive and/or PIMS-Ts	Yes/No
Acute illness including trauma	Yes/No
Routine blood tests	Yes/No
Age <18 years	Yes/No

Exclusion Criteria

Planned admission to PCCU with no acute illness	Yes/No
Declined Consent for research<br>(Or)	Yes/No

For peer review only

Did not respond to follow up letter	
Language barrier cannot understand English/Welsh	Yes/No

Consent: Yes/No

(Please obtain consent before recording further details)

Co-morbidities: *If yes, please specify or give details regarding co-morbidity (prompt in eCRF)*

Congenital Heart disease Yes / No	Chronic Lung disease Yes/No	Neurological Problem Yes/No
Chronic renal disease Yes/No	Gastrointestinal Yes/No	Asthma Yes/No
Diabetes Yes/No	Liver disease Yes/No	Malignancy Yes/No
Exprematurity Yes/No	Haematological problem Yes/No	Immune disorder (including auto-immune and immune deficiency) Yes/No
Other: please specify		

History available for any vaccination in previous 4 weeks: Yes/No

If 'Yes', what vaccination? :

Acute illness onset timeline

Date of onset of symptoms	DD:MM:YY
Date of admission to hospital	DD:MM:YY
Date of admission to ward	DD:MM:YY
Date of admission to PCCU	DD:MM:YY

Please derive relation to research sample and other info such as microbiological tests and outcome variables

Brief history of illness and background

(*please* ***do not*** *include any patient identifiable information such as name, date of birth, age or address***)**

Admission signs and symptoms

Cough Yes/No	Respiratory distress Yes/No	Wheeze Yes/No	Apnoea Yes/No

For peer review only

Runny nose /Rhinorrhoea Yes/No	Sore throat Yes/No	Recessions Yes/No	Lower chest in drawing Yes/No
Grunting Yes/No	Head Bobbing Yes/No	Colour change Yes/No<br>• Specify:	Cardio Respiratory arrest Yes/No
Palpitations Yes/No	Arrhythmia Yes/no	Cool peripheries Yes/No	Thread /weak peripheral pulses Yes/No
Fever Yes/No	Chills/Rigors Yes/No	Seizure Yes/No	Abnormal movement Yes/No
Lethargy/weakness Yes/No	Headache Yes/No	Photophobia Yes/No	Neck stiffness Yes/No
Focal Neurology Yes/No	Poor tone Yes/No	Pupillary abnormalities Yes/No	Altered Consciousness Yes/No<br>• Irritable<br>• Agitation<br>• Abnormal behaviour<br>• Drowsy Yes/No
Vomiting Yes/No<br>• Bilious Vomiting Yes/No<br>• Bloody Vomiting Yes/No	Diarrhoea Yes/No	Bloody stools Yes/No	Constipation Yes/No
Abdominal distension Yes/No	Abdominal pain Yes/No	Reduced feeds Yes/No	Loss of appetite Yes/No
Weight Loss Yes/No	Skin Rash Yes/No<br>• Maculo/popular Yes/No<br>• Petechial/Ecchymosis Yes/No<br>• Vesicular Yes/No<br>• Other - specify	Lymphadenopathy Yes/No	Bleeding Yes/No
Reduced Urine Output Yes/No	Excessive Urination Yes/no	Haematuria Yes/No	Other Please specify

Admitted following unplanned/emergency surgery: Yes/No

For peer review only

If yes: please specify

PCCU Admission vital observations and laboratory details

FiO2(0.21 -1)	**Number 0.XX**
SpO2 %	**Number XXX**
HR beats/min	**NUMBER XXX**
RR Breaths/min	**NUMBER XXX**
BP mm of Hg<br>• Systolic<br>• Diastolic<br>• Mean	**NUMBER XXX**
Temp Centigrade<br>• Core<br>• Peripheral	**NUMBER XX.X**
GCS	**NUMBER XX**
Blood gas<br>• pH<br>• PaO2 KPa<br>• PaCO2 KPa<br>• Base Excess<br>• Bicarbonate mmol/L	**pH NUMBER X.XX**<br>**PaO2 NUMBER XX.XX**<br>**PaCO2 NUMBER XX.XX**<br>**BASE EXCESS NUMBER XX.XX**<br>**BICARBONATE NUMBER XX.X**
Urine output MLS/KG/HR	**NUMBER XX.XX**
Lactate mmol/L	**NUMBER XX.XX**
CRP mg/L	**NUMBER XXX.X**
Beta D Glucan pg/ml	**XXX.X**
Hb g/l	**XX.X**
WCC number / cumm<br>• Lymphocytes<br>• Neutrophils	**NUMBER XXX.X**
Platelets number/cumm	**NUMBER XXXX**
PT seconds	**NUMBER XX**
APTT second	**NUMBER XXX**
INR ratio	**NUMBER X.X**
ALT U/L	**NUMBER XXXXXX**
Bilirubin µmol/L	**NUMBER XX.X**
Urea mmol/L	**NUMBER XX.X**
Creatinine µmol/L	**NUMBER XXX.X**
Na mmol/L	**NUMBER XXX**
K mmol/L	**NUMBER XX.X**
Cl mmol/L	**NUMBER XXX.X**
d-Dimers µg/L	
Ferritin µg/L	**NUMBER XXXX**
CXR Signs of Lower Resp Infection	**Yes/No**
Other<br>• specify	

For peer review only

First observations since onset of acute illness on Ward / presentation to ED (please circle first if Ward or ED) from local or referring hospital.

FiO2(0.21 - 1	**Number 0.XX**
SpO2 %	**Number XXX**
HR beats/min	**NUMBER XXX**
RR Breaths/min	**NUMBER XXX**
BP mm of Hg<br>• Systolic<br>• Diastolic<br>• Mean	**NUMBER XXX**
Temp Centigrade<br>• Core<br>• Peripheral	**NUMBER XX.X**
GCS	**NUMBER XX**
Blood gas<br>• pH<br>• PaO2 KPa<br>• PaCO2 KPa<br>• Base Excess<br>• Bicarbonate mmol/L	**pH NUMBER X.XX**<br>**PaO2 NUMBER XX.XX**<br>**PaCO2 NUMBER XX.XX**<br>**BASE EXCESS NUMBER XX.XX**<br>**BICARBONATE NUMBER XX.X**
Urine output MLS/KG/HR	**NUMBER XX.XX**
Lactate mmol/L	**NUMBER XX.XX**
CRP mg/L	**NUMBER XXX.X**
Beta D Glucanpg/ml	**XXX.X**
Hb g/l	**XX.X**
WCC number / cumm<br>• Lymphocytes<br>• Neutrophils	**NUMBER XXX.X**
Platelets number/cumm	**NUMBER XXXX**
PT seconds	**NUMBER XX**
APTT second	**NUMBER XXX**
INR ratio	**NUMBER X.X**
ALT U/L	**NUMBER XXXXXX**
Bilirubin µmol/L	**NUMBER XX.X**
Urea mmol/L	**NUMBER XX.X**
Creatinine µmol/L	**NUMBER XXX.X**
Na mmol/L	**NUMBER XXX**
K mmol/L	**NUMBER XX.X**
Cl mmol/L	**NUMBER XXX.X**
d-Dimers µg/L	
Ferritin µg/L	**NUMBER XXXX**
CXR Signs of Lower Resp Infection	**Yes / No**
Other<br>• specify	

For peer review only

Paediatric Index of Mortality (PIM-3) score: decimal. 0-1 (range)

Provisional Diagnosis

1. Provisional Diagnosis	**Comments multiple**
2. Provisional Diagnosis	

Organ Support in the first 24 hours

Respiratory Yes/No<br>If 'Yes' tick relevant box to select	• Non Invasive<br>○ High Flow<br>○ CPAP<br>○ BiPAP<br>• Invasive Mechanical ventilation<br>• High Frequency Oscillation	
Cardiovascular<br>Vaso active infusions Yes/No. If 'Yes' ...tick relevant box to select	• Dopamine<br>• Dobutamine<br>• Adrenaline<br>• Noradrenaline<br>• Vasopressin<br>• Milrinone<br>Other – please specify	
Haemofiltration/Haemodialysis Yes/No		
Extra corporeal life support Yes/No		
Worse PELOD score during illness		

1ST Research blood sample at admission

Date / Time to be entered on CRF: DD:MM:YYYY HH:MM

Suspected sepsis /suspected COVID/ non-septic illness

For peer review only

Daily vital observations and laboratory results (worst and best) - please can you make it easy for Awen and Iona to input data as this is data collected for every day of PICU admission of child?

Date of PCCU admission: _ _DD:MM:YY.

To derive relation to time of admission to PCCU, research sample, adverse events and microbiological sample results

FiO2(0.21 -1.0)<br>• highest<br>• lowest	**NUMBER .XX**
SpO2 %<br>• lowest<br>• highest	**NUMBER XXX**
HR beats/min<br>• highest<br>• lowest	**NUMBER XXX**
RR Breaths/min<br>• highest<br>• lowest	**NUMBER XX**
BP mm of Hg<br>• Systolic<br>Lowest in 24 hours<br>Highest in 24 hours<br>• Diastolic<br>Lowest in 24 hours<br>Highest in 24 hours<br>• Mean<br>Lowest in 24 hours<br>Highest in 24 hours	**NUMBER XXX**
Temp Centigrade<br>• Core<br>• Peripheral	**NUMBER XX.X**
GCS<br>lowest<br>highest	**NUMBER XX**

For peer review only - http://bmjopen.bmj.com/site/about/guidelines.xhtml

Blood gas (blood gas parameters in any sample for a 24 hours period)<br>• pH (lowest in 24 hrs)<br>• PaO2 KPa (lowest in 24 hours)<br>• PaCO2 KPa (highest in 24 hours)<br>• Base Excess (from the lowest pH gas result)<br>• Bicarbonate mmol/L (from the lowest pH gas result)	**NUMBERS**<br><br>**pH X.XX**<br>**PaO2 XX.X**<br>**PaCO2 XX.X**<br>**Base Excess XX.X**<br>**Bicarbonate XX.X**
Urine output (UOP)<br>Lowest UOP in any 6 hours over a 24 hours period<br>Diuretic medication Y/N	**NUMBER**<br>**XX.X**
Lactate mmol/L<br>Highest lactate	**NUMBER**<br>**XX.X**
CRP mg/L	**NUMBER XXX.X**
Beta D Glucan pg/ml	**xxx.x**
Hb g/l	**NUMBER XXX**
WCC number / cumm<br>• Lymphocytes<br>• Neutrophils	**NUMBER XXX.X**
Platelets number/cumm	**NUMBER XXXX**
PT seconds	**NUMBER XX**
APTT seconds	**NUMBER XXX**
INR ratio	**NUMBER X.X**
ALT U/L	**NUMBER XXXX**
Bilirubin µmol/L	**NUMBER XXX.X**
Urea mmol/L	**NUMBER XX.X**
Creatinine µmol/L	**NUMBER XXX.X**
Na mmoll/L	**NUMBER XXX**
K mmoll/L	**NUBMER XX.X**
Cl mmoll/L	**NUMBER XXX**
d-Dimers µg/L	**NUMBER XXXXXX**
Ferritin µg/L	**NUMBER XXXXXX**
CXR signs of lower resp infection	**Yes / No**
Other<br>• specify	**entries with free text**

For peer review only

Daily Organ Support 24 hours (00:00 to 24:00) this is done every day, like the information above

Respiratory Yes/No<br>If 'Yes' *tick relevant box below to select*	• Non Invasive<br>○ High Flow<br>○ CPAP<br>○ BiPAP<br>• Invasive Mechanical ventilation<br>• High Frequency Oscillation
Cardiovascular<br>Vaso active infusions Yes/No, if Yes ...tick relevant box	• Dopamine<br>• Dobutamine<br>• Adrenaline<br>• Noradrenaline<br>• Vasopressin<br>• Milrinone<br>Other – please specify
Haemofiltration/Haemodialysis Yes/No	
ECMO Yes/No	

For peer review only

Subsequent Research blood samples: date and time – DD:MM:YY HH:MM

To derive duration from time of admission: __:__ (hh:mm) and other events such as death and blood cultures

Time point (tp1 to tp7)	Date and time	Clinical Correlation eg: admission; onset of hypotension, cardiac arrest etc.,

Procedures during admission:

Endotracheal Intubation: Y/N

Central Venous Line Insertion: Y/N

Arterial Line Insertion: Y/N

Chest Drain insertion: Y/N

Date of procedure: DD/MM/YYYY

Other:

Please specify

Surgery during PCCU admission: Yes/No DATE TIME DD:MM:YY HH:MM

If yes, please specify:

DATE AND TIME DD:MM:YY HH:MM

To derive relation to admission to PCCU and research sample date and time

Microbiological investigations (includes bacterial, viral, fungal or any other investigations to identify a causative organism for illness)

1. Blood culture: *Provision to input more than one entry for all categories from 1 to 8*

For peer review only

Requested Y/N

If Y, Result: Positive/Negative

If 'Positive' - please specify

If positive is this significant or no significant? Check box.

Free text for the bacterium

DATE AND TIME DD:MM:YY HH:MM

To derive relation to research sample date and time

2. Naso-pharyngeal aspirate for respiratory panel:

Requested: Yes/No

If yes, result:

Influenza A PCR RNA
Influenza B PCR RNA
RSV PCR RNA
Parainfluenza PCR RNA
Rhinovirus/Enterovirus PCR
HMPV PCR RNA
Adenovirus PCR DNA
Mycoplasma PCR DNA
Enterovirus PCR RNA
Coronavirus PCR

Positive/Negative

If 'Positive' – please specify

DATE AND TIME DD:MM:YY HH:MM

To derive relation to research sample date and time

3. COVID-19 test:

Requested: Yes/No

Throat swap: Yes/No

If Yes- Positive/Negative?

non Bronchoscopic Alveolar Lavage (nBAL): Yes/No

If 'Yes' – Positive/Negative?

DATE AND TIME DD:MM:YY HH:MM

To derive relation to research sample date and time

For peer review only

4. Urine MCS

Requested: Yes/No

If yes, result: Positive/Negative

If 'Positive' – please specify

DATE AND TIME DD:MM:YY HH:MM

To derive relation to research sample date and time

5. CSF MCS

Requested: Yes/No

If yes, result: Positive/Negative

If 'Positive' – please specify

DATE AND TIME DD:MM:YY HH:MM

To derive relation to research sample date and time

6. Non Bronchoscopy Alveolar Lavage (NBAL)

Requested: Yes/No

If yes, result: Positive/Negative

If 'Positive' – please specify

DATE AND TIME DD:MM:YY HH:MM

To derive relation to research sample date and time

7. Sputum culture

Requested: Yes/No

If yes, result: Positive/Negative

If 'Positive' – please specify

DATE AND TIME DD:MM:YY HH:MM

To derive relation to research sample date and time

8. Other specimens/Tests

Please specify

For peer review only

(Prompt to add multiple distinct entries)

DATE AND TIME DD:MM:YY HH:MM

To derive relation to research sample date and time

Medications during PCCU admission:

1. Corticosteroid: Yes/No
   If 'Yes', please tick relevant box/boxes to select
   Prednisolone
   Methylprednisolone
   Hydrocortisone
   Dexamethasone

   Other: *please specify*
   Indication

   Dose (Mg/Kg)

   Date started DD/MM/YY
   Date stopped DD/MM/YY
   To derive duration and relation to date of admission to PCCU

2. Antibiotic: Yes/No
   If 'Yes', please tick relevant box/boxes to select

   Co-Amoxiclavulonic acid (Augmentin)
   Cefotaxime
   Ceftriaxone
   Tazocin
   Meropenem
   Azithromycin
   Clarithyromycin

   Other*: please specify*

   Indication:
   Date started: DD:MM:YY
   Date stopped: DD:MM:YY
   To derive duration and relation to date of admission to PCCU

3. Antiviral Yes/No
   If yes, please tick relevant box/boxes to select

For peer review only

Aciclovir
Gancliclovir

Other: *please specify*

Indication:

Date started: DD/MM/YY
Date stopped: DD/MM/YY
To derive duration and relation to date of admission to PCCU

4. Antifungal Y/N
   If 'Yes', please tick relevant box/boxes to select

   Fluconazole
   Ambisome
   Itraconazole

   Other: please specify

   Indication:

   Date started: DD/MM/YY
   Date stopped: DD/MM/YY
   To derive duration and relation to date of admission to PCCU

5. Anti-inflammatory medications : Yes/No
   If 'Yes', please tick relevant box/boxes to select

   Ibuprofen
   Other NSAID
   Other: please specify

   Indication:

   Date started: DD/MM/YY
   Date stopped: DD/MM/YY
   To derive duration and relation to date of admission to PCCU
6. Other medications modifying immunity :: Yes/No
   (Includes chemotherapy or Antibody based medications)
   If 'Yes', please tick relevant box/boxes to select

   Intravenous Immunoglobulin (IVIG)
   Methotrexate
   Vincristine
   Daunorubicin

For peer review only

Infliximab
Anakinra
Tocilizumab

Other: please specify

Indication:

Date Started: DD/MM/YY

Date Stopped: DD/MM/YY

To derive duration and relation to date of admission to PCCU

Clinical Outcome:

1. Duration on mechanical ventilation: __(DD)
2. Duration on Vase-active agents:__(DD)
3. Date of discharge from PCCU: DD/MM/YY
   To derive duration of stay in PCCU: ___ (DD)
4. Alive at discharge: Yes/No
   If No to 4, (clinical outcome is death), Date of death: DD/MM/YY
   To derive duration of stay in PCCU
   To derive duration from research blood samples
   Withdrawal of life support: Yes/No?
5. Transfer to different hospital: Y/N

Confirmed diagnosis at time of discharge from PCCU: Yes/No

(In case of infectious illness – please specify if microbiologically confirmed)

If 'Yes' please specify

(Please include microbiological diagnosis as well as the complications e.g.: exacerbation of asthma secondary to Rhinovirus infection)

This may involve conclusion following Multi-professinal discussions at the time including Infectious Disease specialist, Microbiologist or any other specialists.

Indeterminate diagnosis at discharge from PCCU: Yes/No

:

Final diagnosis available from clinical follow up Yes/No

If 'Yes' please give details

Is the Adjudicator's diagnosis same as the final diagnosis on follow up?

Yes/No

For peer review only

Clinical Phenotyping

Consensus between two clinicians? Yes /No

If no..

FINAL ADJUDICATOR OPINION:

if yes..

CONSENSUS CLINICAL PHENOTYPING:

Infectious Illness: Yes ☐ No ☐

If infectious illness..

please choose:

- Suspected ☐ (or)
- Proven by positive culture, tissue stain, or PCR test ☐ (or)
- a clinical syndrome associated with a high probability of infection ☐

Type of infectious illness:

Not sepsis ☐

Sepsis ☐

If sepsis..

Choose microbiological agent:

- Sepsis with confirmed bacterial infection ☐
- Sepsis with confirmed other microbiological (eg. Viral, fungal) infection ☐
- Sepsis with no microbiological confirmation ☐

Choose severity of sepsis:

- Sepsis ☐
- Sepsis associated organ dysfunction cardiovascular or non-cardiovascular organ dysfunction ☐
- Septic shock ☐

Diagnosis

Non-infectious illness – please state diagnosis ______________________

Sterile inflammatory illness ☐ eg. Trauma (or) Asthma

Non inflammatory illness ☐ eg. DKA or Status Epilepticus

Infectious illness – please state diagnosis _______________________

For peer review only

Adverse events: Yes/No

If 'Yes', please specify details:

Date and Time: DD/MM/YY

(DATE FOR USE ONLY BY SPONSOR)

Please derive duration from admission to PCCU

Was it related to research?

If yes, please specify if expected from research procedures or unexpected

Was the adverse event considered serious? : Yes/No

If yes, please specify details why it was considered serious:

Has the SAE (related) been reported to REC/Sponsor: Yes/No

(Deaths or complications from acute illness are not considered serious adverse events)

For peer review only

Case Report Form (CRF) review tool for clinical phenotyping

Criteria for sepsis

Paediatric consensus conference sepsis criteria, 2005

Presence of Systemic Inflammatory Response Syndrome with suspected or proven infection.

Description of criteria for the PCCS definition of sepsis:

<u>Systemic Inflammatory Response Syndrome (SIRS)</u>

The presence of at least two of the following four criteria, one of which must be abnormal temperature or leukocyte count: This is during any point from first contact with health care professionals or during stay in PCCU.

- Core temperature >38.5C or <36C ☐
- Tachycardia, defined as a mean heart rate of >2SD above normal for age ☐
  Or (for infants, bradycardia < 10th centile for age)
- Mean respiratory rate > 2SD above normal for age or mechanical ventilation for an acute process ☐
- Leucocyte count elevated or depressed for age ☐

Evidence of infection includes positive findings on clinical exam, imaging, laboratory tests (e.g., white blood cells in a normally sterile body fluid, perforated viscus, chest radiograph consistent with pneumonia, petechial or purpuric rash, or purpura fulminans)

Age specific vital signs and laboratory parameters:

Age Group[a]	Heart Rate, Beats/Min[b,c]		Respiratory Rate, Breaths/Min[d]	Leukocyte Count, Leukocytes × 10^3/mm[3b,c]	Systolic Blood Pressure, mm Hg[b,c,e,f]
	Tachycardia	Bradycardia			
0 days to 1 wk	>180	<100	>50	>34	<65
1 wk to 1 mo	>180	<100	>40	>19.5 or <5	<75
1 mo to 1 yr	>180	<90	>34	>17.5 or <5	<100
2–5 yrs	>140	NA	>22	>15.5 or <6	<94
6–12 yrs	>130	NA	>18	>13.5 or <4.5	<105
13 to <18 yrs	>110	NA	>14	>11 or <4.5	<117

Surviving sepsis campaign for sepsis guidelines in children, 2020

- **Defined as per the PCCS 2005 guideline (OR) evidence of *severe bacterial infection leading to life threatening organ dysfunction would be included, even when there is deviation from the specific 2005 criteria above.***

For peer review only - http://bmjopen.bmj.com/site/about/guidelines.xhtml

<u>Study ID:</u>

Consensus between two clinicians Yes /No

If ‘No’

Final Adjudicator opinion sought Yes/No

Acute illness: Yes ☐ No ☐

Infectious Illness: Yes ☐ No ☐

If infectious illness, please state:

- Suspected ☐ (or)
- Proven by positive culture, tissue stain, or PCR test ☐ (or)
- a clinical syndrome associated with a high probability of infection ☐

Type of infectious illness:

Not sepsis ☐

Sepsis ☐ If sepsis, please choose one below

- Sepsis with confirmed bacterial infection ☐
- Sepsis with confirmed viral infection ☐
- Sepsis with no microbiological confirmation ☐

Severity of sepsis is defined based on organ function in to

- Sepsis ☐
- Sepsis associated organ dysfunction cardiovascular or non-cardiovascular organ dysfunction ☐
- Septic shock – sepsis with cardiovascular organ dysfunction with evidence of hypotension, need for treatment with vasoactive medication or impaired perfusion. ☐

Diagnosis

Non-infectious illness – please state diagnosis ______________________

Sterile inflammatory illness ☐ eg. Trauma (or) Asthma

Non inflammatory illness ☐ eg. Diabetic Ketoacidosis or prolonged febrile seizure

Infectious illness – please state diagnosis _______________________

Comments:

For peer review only - http://bmjopen.bmj.com/site/about/guidelines.xhtml

3

For peer review only

Clinical phenotyping of patients recruited to pSeP/COVID study
Decision flow chart for reviewers
v 0.1

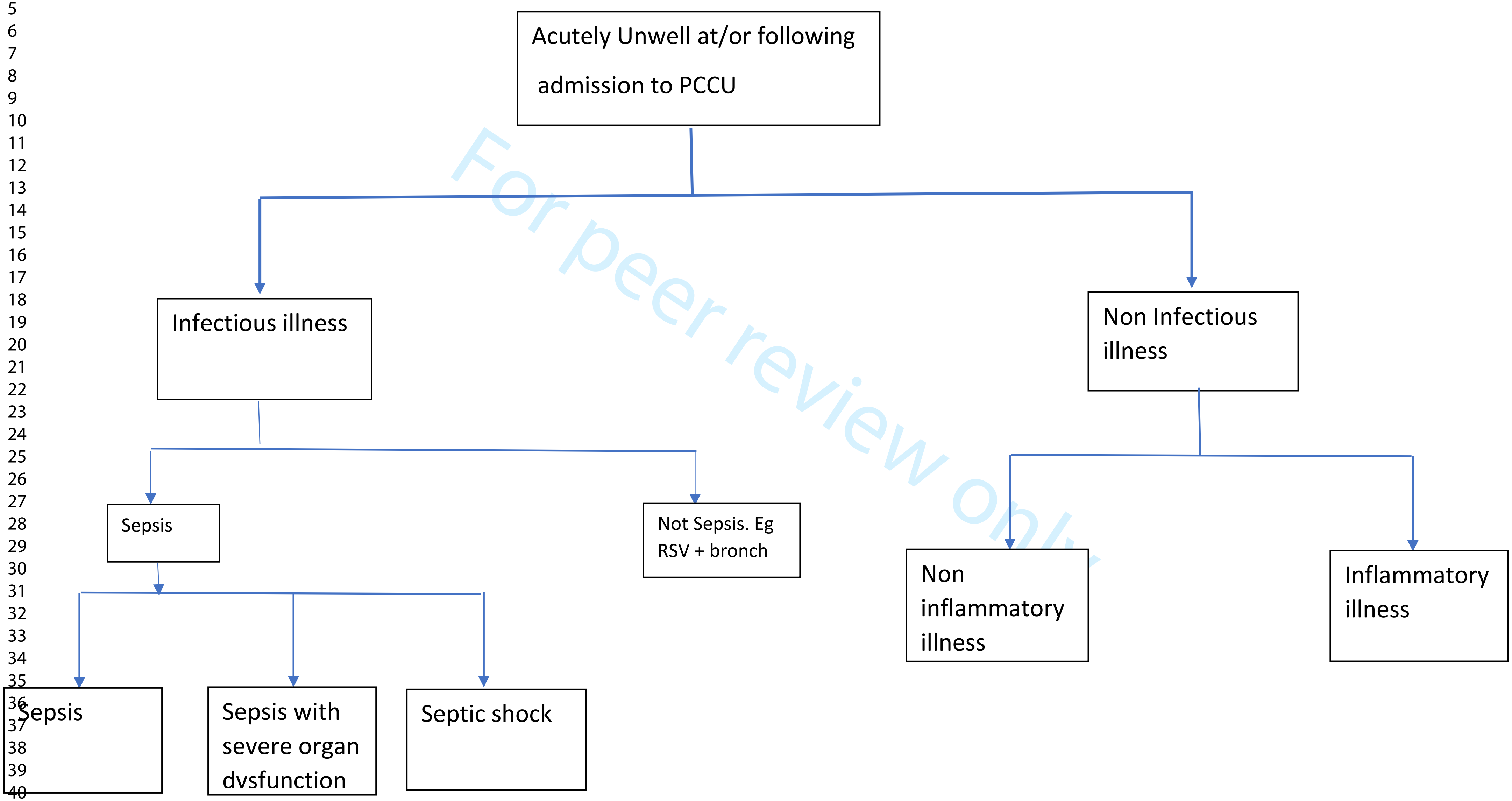

For peer review only

Immune and metabolic markers for identifying and investigating severe Coronavirus disease and Sepsis in children and young people. (pSeP/COVID ChYP study) – Protocol for a prospective cohort study

STROBE Statement—checklist of items that should be included in reports of observational studies

	Item No.	Recommendation	Page No.	Relevant text from manuscript
**Title and abstract**	1	(*a*) Indicate the study's design with a commonly used term in the title or the abstract	1	Included
		(*b*) Provide in the abstract an informative and balanced summary of what was done and what was found		
**Introduction**				
Background/rationale	2	Explain the scientific background and rationale for the investigation being reported		Included under Introduction
Objectives	3	State specific objectives, including any prespecified hypotheses		Included under Methods
**Methods**				
Study design	4	Present key elements of study design early in the paper		Included in Methods
Setting	5	Describe the setting, locations, and relevant dates, including periods of recruitment, exposure, follow-up, and data collection		Included in the Methods
Participants	6	(*a*) *Cohort study*—Give the eligibility criteria, and the sources and methods of selection of participants. Describe methods of follow-up<br>*Case-control study*—Give the eligibility criteria, and the sources and methods of case ascertainment and control selection. Give the rationale for the choice of cases and controls<br>*Cross-sectional study*—Give the eligibility criteria, and the sources and methods of selection of participants		Included in Methods<br>For nested case-control analysis, provided details of the Clinical phenotyping to ascertain cases and controls
		(*b*) *Cohort study*—For matched studies, give matching criteria and number of exposed and unexposed<br>*Case-control study*—For matched studies, give matching criteria and the number of controls per case		N/A
Variables	7	Clearly define all outcomes, exposures, predictors, potential confounders, and effect modifiers. Give diagnostic criteria, if applicable		Provided the details of variables to be measured. Discussed the process for clinical phenotyping

For peer review only - http://bmjopen.bmj.com/site/about/guidelines.xhtml

Immune and metabolic markers for identifying and investigating severe Coronavirus disease and Sepsis in children and young people. (pSeP/COVID ChYP study) – Protocol for a prospective cohort study

Data sources/ measurement	8*	For each variable of interest, give sources of data and details of methods of assessment (measurement). Describe comparability of assessment methods if there is more than one group	Provided details of the sample collection, processing and collection of data. Also provided template of Case Report Form which is used to collect clinical data.
Bias	9	Describe any efforts to address potential sources of bias	Discussed relevant bias and measures to address in the Methods.
Study size	10	Explain how the study size was arrived at	Provided details under section Sample Analysis, Sample size calculation

Continued on next page

For peer review only

Immune and metabolic markers for identifying and investigating severe Coronavirus disease and Sepsis in children and young people. (pSeP/COVID ChYP study) – Protocol for a prospective cohort study

Quantitative variables	11	Explain how quantitative variables were handled in the analyses. If applicable, describe which groupings were chosen and why	Provided the relevant information for the analysis under section Clinical Phenotyping and Sample Analysis
Statistical methods	12	(*a*) Describe all statistical methods, including those used to control for confounding	Provided information in section Methods, Data processing and analysis
		(*b*) Describe any methods used to examine subgroups and interactions	
		(*c*) Explain how missing data were addressed	
		(*d*) *Cohort study*—If applicable, explain how loss to follow-up was addressed<br>*Case-control study*—If applicable, explain how matching of cases and controls was addressed<br>*Cross-sectional study*—If applicable, describe analytical methods taking account of sampling strategy	As the study is aimed to evaluate biomarkers in blood samples, this has not been addressed.
		(*e*) Describe any sensitivity analyses	
**Results**			
Participants	13*	(a) Report numbers of individuals at each stage of study—eg numbers potentially eligible, examined for eligibility, confirmed eligible, included in the study, completing follow-up, and analysed	Not Applicable
		(b) Give reasons for non-participation at each stage	Not Applicable
		(c) Consider use of a flow diagram	Not Applicable
Descriptive data	14*	(a) Give characteristics of study participants (eg demographic, clinical, social) and information on exposures and potential confounders	Not Applicable
		(b) Indicate number of participants with missing data for each variable of interest	Not Applicable
		(c) *Cohort study*—Summarise follow-up time (eg, average and total amount)	Not Applicable
Outcome data	15*	*Cohort study*—Report numbers of outcome events or summary measures over time	*Not Applicable*
		Case-control study—Report numbers in each exposure category, or summary measures of exposure	*Not Applicable*
		Cross-sectional study—Report numbers of outcome events or summary measures	*Not Applicable*

For peer review only - http://bmjopen.bmj.com/site/about/guidelines.xhtml

Immune and metabolic markers for identifying and investigating severe Coronavirus disease and Sepsis in children and young people. (pSeP/COVID ChYP study) – Protocol for a prospective cohort study

Main results	16	(*a*) Give unadjusted estimates and, if applicable, confounder-adjusted estimates and their precision (eg, 95% confidence interval). Make clear which confounders were adjusted for and why they were included	Not Applicable
		(*b*) Report category boundaries when continuous variables were categorized	Not Applicable
		(*c*) If relevant, consider translating estimates of relative risk into absolute risk for a meaningful time period	Not Applicable

Continued on next page

For peer review only

Immune and metabolic markers for identifying and investigating severe Coronavirus disease and Sepsis in children and young people. (pSeP/COVID ChYP study) – Protocol for a prospective cohort study

Other analyses	17	Report other analyses done—eg analyses of subgroups and interactions, and sensitivity analyses	Not Applicable
**Discussion**			
Key results	18	Summarise key results with reference to study objectives	Not Applicable
Limitations	19	Discuss limitations of the study, taking into account sources of potential bias or imprecision. Discuss both direction and magnitude of any potential bias	Not Applicable
Interpretation	20	Give a cautious overall interpretation of results considering objectives, limitations, multiplicity of analyses, results from similar studies, and other relevant evidence	Not Applicable
Generalisability	21	Discuss the generalisability (external validity) of the study results	Not Applicable
**Other information**			
Funding	22	Give the source of funding and the role of the funders for the present study and, if applicable, for the original study on which the present article is based	Not Applicable

*Give information separately for cases and controls in case-control studies and, if applicable, for exposed and unexposed groups in cohort and cross-sectional studies.

Note: An Explanation and Elaboration article discusses each checklist item and gives methodological background and published examples of transparent reporting. The STROBE checklist is best used in conjunction with this article (freely available on the Web sites of PLoS Medicine at http://www.plosmedicine.org/, Annals of Internal Medicine at http://www.annals.org/, and Epidemiology at http://www.epidem.com/). Information on the STROBE Initiative is available at www.strobe-statement.org.

For peer review only
